# Liraglutide Improves Cognitive and Neuronal Function in 3-NP Rat Model of Huntington’s Disease

**DOI:** 10.3389/fphar.2021.731483

**Published:** 2021-12-22

**Authors:** Samar M. Shawki, Mohammed A. Saad, Rania M. Rahmo, Walaa Wadie, Hanan S. El-Abhar

**Affiliations:** ^1^ Department of Pharmacology and Toxicology, Faculty of Pharmacy, Misr International University, Cairo, Egypt; ^2^ Department of Pharmacology and Toxicology, Faculty of Pharmacy, Cairo University, Cairo, Egypt; ^3^ School of Pharmacy, Newgiza University, Cairo, Egypt; ^4^ Department of Pharmacology, Toxicology and Biochemistry, Faculty of Pharmacy, Future University in Egypt, Cairo, Egypt

**Keywords:** Huntington’s disease, 3-nitropropionic acid, (BDNF)/(TrkB), neurotrophic, liraglutide

## Abstract

Huntington’s disease (HD) is an autosomal dominant inherited neurodegenerative disease characterized by progressive motor, psychiatric, and cognitive abnormalities. The antidiabetic drug liraglutide possesses a neuroprotective potential against several neurodegenerative disorders; however, its role in Huntington’s disease (HD) and the possible mechanisms/trajectories remain elusive, which is the aim of this work. Liraglutide (200 μg/kg, s.c) was administered to rats intoxicated with 3-nitropropionic acid (3-NP) for 4 weeks post HD model induction. Liraglutide abated the 3-NP-induced neurobehavioral deficits (open field and elevated plus maze tests) and histopathological changes. Liraglutide downregulated the striatal mRNA expression of HSP 27, PBR, and GFAP, while it upregulated that of DARPP32. On the molecular level, liraglutide enhanced striatal miR-130a gene expression and TrKB protein expression and its ligand BDNF, while it reduced the striatal protein content and mRNA expression of the death receptors sortilin and p75NTR, respectively. It enhanced the neuroprotective molecules cAMP, p-PI3K, p-Akt, and p-CREB, besides modulating the *p*-GSK-3β/*p*-β-catenin axis. Liraglutide enhanced the antioxidant transcription factor Nrf2, abrogated TBARS, upregulated both Bcl2 and Bcl-XL, and downregulated Bax along with decreasing caspase-3 activity. Therefore, liraglutide exerts a neurotherapeutic effect on 3-NP-treated rats that is, besides the upturn of behavioral and structural findings, it at least partially, increased miR-130a and modulated PI3K/Akt/CREB/BDNF/TrKB, sortilin, and p75NTR, and Akt/GSK-3β/*p*-β-catenin trajectories besides its capacity to decrease apoptosis and oxidative stress, as well as its neurotrophic activity.

## 1 Introduction

Huntington’s disease (HD) is an autosomal dominant, progressive, and devastating neurodegenerative disorder caused by a polyglutamine expansion in exon 1 of the huntingtin gene (Htt) with the primary site of neuron loss is at the striatal part of the basal ganglia (Agrawal and Fox, 2019; Lallani et al., 2019). HD is a rare disease that mainly affects 5–7 individuals per 100,000 population associated with significant morbidity and mortality (Bruzelius et al., 2019). The hallmark features of HD are the presence of abnormal involuntary jerking and writhing movements (progressive chorea) accompanied by cognitive decline. As a consequence, HD patients are burdened with uncontrolled motor deficits, cognitive impairments, and neuropsychiatric symptoms (Huguet et al., 2019). Altered delivery of brain-derived neurotrophic factor (BDNF) to the striatum and consequently its diminished striatal levels are believed to underlie the high vulnerability of the striatum to neuronal loss in HD (Suelves et al., 2019).

3-Nitropropionic acid (3-NP) is a mitochondrial toxin used to induce HD-like symptoms in rats. This toxin irreversibly inhibits the enzyme succinate dehydrogenase (complex II) of the electron transport chain and Kreb’s cycle leading to selective massive loss of GABAergic medium-sized spiny neurons (MSNs) of the striatum, i.e., the typical pathology observed in HD (Cirillo et al., 2019). Despite previous studies, Durães et al., (2018) and Barker and Mason (2019) have stated that HD is still incurable with very limited therapies that target the symptoms only, and the available medications can only lessen some motor, cognitive, and psychiatric symptoms. Another work (Tabrizi et al., 2019) has recounted that strategies that aim at decreasing huntingtin are able to modify HD, a notion that emphasizes the importance of treating the causes of HD rather than mitigating only its symptoms.

Incretin hormones are a group of metabolic hormones, which are rapidly inactivated by dipeptidyl peptidase-4 (DPP-4). One incretin of particular concern is the glucagon-like peptide-1 (GLP-1) that can primarily cross the blood–brain barrier (BBB) to influence quite a lot of cellular pathways within the central nervous system (CNS). Existing research recognizes the critical neurotrophic and neuroprotective effects of GLP-1 on increasing the level of the BDNF in the cortex, hippocampus, and striatum to protect against neuronal apoptosis and improve the neuronal differentiation (Bomba et al., 2018). Hence, the implication of GLP-1 receptors (GLP-1Rs) may afford a promising target for the treatment of HD disease (Yang et al., 2017a).

To overcome the drawback of the short half-life of endogenous GLP-1, several DPP-4 resistant GLP-1 analogs were developed including liraglutide (Lira). Lira is an analog of the endogenous human GLP-1 with 97% of sequence homology (Sharma et al., 2018). Lira is an approved antidiabetic drug that acts as an incretin mimetic GLP-1R agonist and is increasingly used to improve glycemic control, reduce the risk of heart attack, stroke, and cardiovascular death in adults with type 2 diabetes mellitus (T2DM) (Marso et al., 2016). Besides its documented antidiabetic effect, Lira possesses an extrapancreatic neuroprotective potential against models of Parkinson’s disease (Badawi et al., 2017), Alzheimer’s disease (Zhang et al., 2019a), stroke (Zhao et al., 2018), and diabetes-associated neurodegeneration (Filchenko et al., 2018); however, its possible role in HD models has yet to be determined.

Based on the aforementioned data, we attempt to assess the effect of Lira on the 3-NP-induced HD-like mode through its ability to recover altered cognition and behavior, to restore striatal morphology, and explore some trajectories that can be involved in its therapeutic effect.

## 2 Materials and Methods

### 2.1 Animals

Adult male Wistar rats weighing 250 ± 20 g were obtained from the National Research Centre (NRC, Giza, Egypt) and were allowed to acclimatize in the animal facility of the Faculty of Pharmacy (Cairo University) for a week prior to starting any experimental procedure. Rats were housed under controlled environmental conditions of constant temperature (25 ± 2°C), on a 12/12-h light/dark cycle. Rats were permitted free access to standard food and water *ad libitum*, and all behavioral tests were carried out in a sound isolated laboratory. The protocols used in this study complied with “The Guide for Care and Use of Laboratory Animals” published by the US National Institutes of Health (NIH publication no. 85–23, revised 2011) and were approved by the Ethics Committee for Animal Experimentation at Faculty of Pharmacy, Cairo University (permit number: PT 2099). All efforts were made to minimize animal suffering and to reduce the number of animals used.

### 2.2 Drugs and Chemicals

3-NP was purchased from Sigma-Aldrich (St. Louis, MO, USA), freshly prepared daily in normal saline, and neutralized to pH 7.4 with NaOH. It was administered through intraperitoneal (i.p.) route to each animal at a dose of 10 mg/kg body weight. The selection of dose and duration of 3-NP administration was based on published studies (El-Abhar et al., 2018). Liraglutide (Lira) was purchased as a raw material from Sigma-Aldrich Chemical Co. (St. Louis, MO, USA), and the dose was chosen after performing a pilot study in which three different dose levels (50, 100, and 200 μg/kg/day) were injected subcutaneously (s.c.) for 4 weeks (Dong et al., 2017), and different parameters were assessed to aid in dose selection; *viz.*, neurocognitive behavioral tests, body weight, histopathological changes, and striatal contents of phosphorylated cAMP response element-binding protein (p-CREB) and BDNF. Since BDNF can be detected in several brain regions, namely, the cortex, striatum, and hippocampus, the content of BDNF was assessed in these areas in the different groups. According to the results of the pilot study (data not shown) a dose of 200 μg/kg and the striatal area were used for the core study parameters. The concentration of the drugs in the prepared solutions was adjusted, so that the required dose for each 200 g rat was found in 2 ml. All other reagents used were of analytical grade.

### 2.3 Experimental Design

**FIGURE 1:**
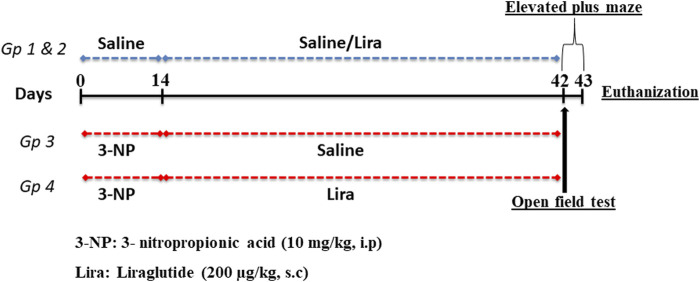
Schematic presentation of the experimental design. The 3-nitropropionic acid (3-NP) (10 mg/kg, i.p.) was injected daily for 14 days, followed by 4 weeks of daily administration of liraglutide (200 μg/kg, s.c.). On days 42 and 43, animals were subjected to behavioral tests and killed on day 43.

As depicted in Figure 1, 60 rats were randomly allocated into one of four treatment groups (*n* = 15) by a technical assistant who was not involved in the analysis. Animals in groups 1 and 2 were injected with normal saline (i.p.) for 14 days, this was followed by s.c administration of saline or Lira, respectively, for another 28 days. In groups 3 and 4, rats received 3-NP (10 mg/kg dissolved in 0.9% saline; i.p.) for 14 days, followed by an s.c injection of either normal saline or Lira for another 28 days. At the end of the study period, all animals were subjected to behavioral tests that were arranged from the least stressful (open-field test; OFT) to the more stressful (elevated plus maze test; EPM) test with a 2-h rest period between the two tests. All behavioral tests were carried out in an attenuated sound testing room with dim light starting at 6.00 p.m., during the light cycle of the animal.

Afterward, the rats in each group were divided into three subsets and then euthanized by an overdose of thiopental (100 mg/kg). The brains were then rapidly harvested, and the striata were dissected out and stored at −80°C until analysis using ELISA (*n* = 6), Western blot (*n* = 3), and RT-qPCR (*n* = 3). For the histopathological study, the whole brains of the other three representative animals were fixed in 10% (v/v) formalin.

### 2.4 Assessment of Parameters

#### 2.4.1 Body Weight

The body weight was measured on the first and last days of the experiment, and the percentage change was calculated in comparison with the initial measurement on the first day as follows:

% Change in body weight = [(weight on day 42 - initial weight)/ initial weight] × 100

#### 2.4.2 Behavioral Assessments

##### 2.4.2.1 Open Field test

The spontaneous locomotor behaviors were assessed using the OFT in which each rat was placed individually into the center of the open field apparatus composed of a square black wooden box (80 × 80 × 40 cm) with smooth polished floor divided into 16 squares 4 × 4 by yellow lines. Summarily, each animal was gently placed in the center of the open field and remote monitored using an overhead camera for 5 min. The apparatus floor and walls were wiped and cleaned after each tested animal with a wet sponge and a dry paper towel to eliminate possible bias due to odors left by previous rats. During the 5-min period, the latency in leaving the starting square and the ambulation (number of squares crossed), grooming, and rearing (number of attempts the animal stretched on its hind limbs with or without forelimb support) frequencies were measured. A line crossing was counted when all four paws crossed over the line (Thangarajan et al., 2016).

##### 2.4.2.2 Elevated Plus Maze Test

Rats were subjected to the EPM using the method described by Kumar et al. (2017) to assess memory retention and cognitive function of the animals. The apparatus consisted of two opposite open arms (50 × 10 cm), crossed with two closed arms of the same dimensions with 40-cm-high walls, connected by a central square (10 × 10 cm); the maze was elevated to a height of 50 cm from the floor. On the first day, each rat was placed individually at one end of an open arm, and the time taken by the rat to move from the open arm to one of the closed arms was recorded as the initial transfer latency (ITL). The rat was allowed to explore the maze for 30 s before being returned to its home cage. In case the animal failed to enter the closed arm within 90 s, the animal was gently pushed into the closed arm, and the ITL for that animal was assigned as 90 s. The retention latency was noted again on the following day, and the percent retention of memory was calculated by the following formula:

[Transfer latency(day 42 - day 43)/Transfer latency(day 42)] × 100

#### 2.4.3 Biochemical Measurements

##### 2.4.3.1 Parameters Assessed by ELISA

The ELISA kits purchased from MyBiosource, Inc. (Southern California, San Diego, CA, United States) were used for estimating the striatal contents of cyclic adenosine monophosphate (cAMP; cat#: MBS018906), BDNF (cat#: MBS355345), and nuclear factor E2-related factor 2 (Nrf-2; cat#: MBS752046), whereas striatal content of thiobarbituric acid reactive substances (TBARS) was measured using a rat ELISA kit (cat#:MD 25 28) purchased from Bio-Diagnostics (Worcestershire, UK). For the determination of caspase-3 activity (cat# ab39401), the corresponding ELISA kit was purchased from Abcam (Cambridge, UK). For all parameters, the assays were performed according to the protocol of the manufacturer. The protein content was quantified according to the method described by Bradford (Camby et al., 2002).

##### 2.4.3.2 Quantitative Real-Time Polymerase Chain Reaction

Total RNA was extracted and purified from the homogenized rat striatal tissue using Qiagen tissue extraction kit (Qiagen, USA), and the purity (A260/A280 ratio) and the concentration of RNA were obtained using spectrophotometry (dual wavelength Beckman, Spectrophotometer, USA). The total RNA (0.5–2 μg) was used for cDNA conversion using high-capacity cDNA reverse transcription kit (Fermentas, USA) according to the instructions of the manufacturer. Quantitative RT-PCR was performed to assess the gene expression of the glial fibrillary acidic protein (GFAP), heat shock protein 27 (HSP27), dopamine- and cAMP-regulated phosphoprotein (DARPP-32), peripheral benzodiazepine receptor (PBR), p75 neurotrophin receptor (p75NTR), BCL2-associated X (Bax), B-cell lymphoma 2 (Bcl2), B-cell lymphoma-extra large (Bcl-XL), and miR-130a using SYBR Green I. The qPCR amplification and analysis were performed using an Applied Biosystem with software version 3.1 (StepOne™, USA) according to the instructions of the manufacturer. Briefly, 3 μl of random primers (Table 1) were added to the 10 μl of RNA, which was denatured for 5 min at 65°C in the thermal cycler. The RNA primer mixture was cooled to 4°C, and the cDNA master mix was prepared according to the kit instructions and added (19 μl) to each sample; this was added to the 13 μl of RNA-primer mixture resulting in 50 μl of cDNA. The last mixture was incubated in the programmed thermal cycler for 1 h at 37°C followed by inactivation of enzymes at 95°C for 10 min and finally cooled at 4°C, then RNA was changed into cDNA. The converted cDNA was stored at −20°C. The relative quantitation (RQ) of the target genes was calculated using the 2−∆∆Ct formula (Livak and Schmittgen, 2001). All values were normalized to the housekeeping gene β-actin, except for miR-130a that was normalized to U6, and presented as fold changes.

**TABLE 1:** The primer sequences used for RT-qPCR.

Gene	Primer sequence (5′–3′)
miR-130a	Forward: 5′-ACA​CTC​CAG​CTG​GGG​CTC​TTT​TCA​CAT​TGT-3′
	Reverse: 5′-CTC​AAC​TGG​TGT​CGT​GGA​GTC​GGC​AAT​TCA​GTT​GAG​AGT​AGC​AC-3′
U6	Forward: 5′-CTC​GCT​TCG​GCA​GCA​CA-3′
	Reverse: 5′-AAC​GCT​TCA​CGA​ATT​TGC​GT-3′
Glial fibrillary acidic protein (GFAP)	Forward: 5′- GCT​AAT​GAC​TAT​CGC​CGC​CAA​CT-3′
	Reverse: 5′-CTC​CTT​AAT​GAC​CTC​GCC​ATC​CC-3′
Heat shock protein 27 (HSP27)	Forward: 5′-ACG​AAG​AAA​GGC​AGG​ATG​AA-3′
	Reverse: 5′-GCT​CCA​GAC​TGT​TCC​GAC​TC-3′
Dopamine- and cAMP-regulated phosphoprotein (DARPP-32)	Forward: 5′-AGT​TAG​GGG​AGC​TTC​G-3′
	Reverse: 5′-AGT​TTC​CAT​CTC​TCT​GGG-3′
Peripheral benzodiazepine receptor (PBR_	Forward: 5′-TCC​TGC​TTT​CAT​GAC​CAT​TGG​GC-3′
	Reverse: 5′-ACA​ACT​GTC​CCC​GCA​TGG​GAC​TTA​G-3′
p75 neurotrophin receptor (p75NTR)	Forward: 5′-AGG​GAT​GGC​GTG​ACT​TTC-3′
	Reverse: 5′-GTT​GGC​TTC​AGG​CTT​ATG​C-3′
B-cell lymphoma 2-associated X protein (Bax)	Forward: 5′-CCC​TGT​GCA​CTA​AAG​TGC​CCC-3′
	Reverse: 5′-GTC​AGA​TGG​ACA​CAT​GGT​G-3′
B-cell lymphoma 2 (Bcl2)	Forward: 5′-CTA​CGA​GTG​GGA​TGC​TGG​AGG-3′
	Reverse: 5′-GTC​AGA​TGG​ACA​CAT​GGT​G-3′
B-cell lymphoma-extra large (Bcl-XL)	Forward: 5′-GAT​CCC​CAT​GGC​AGC​AGT​AAA​GCA​AG-3′
	Reverse: 5′- CCC​CAT​CCC​GGA​AGA​GTT​CAT​TCA​CT-3′
β-Actin	Forward: 5′-GGT​CGG​TGT​GAA​CGG​ATT​TGG-3′
	Reverse: 5′-ATG​TAG​GCC​ATG​AGG​TCC​ACC-3′

##### 2.4.3.3 Western Blot Analysis

After proteins were extracted from striatal tissues using RIPA lysis buffer PL005, equal amounts of the samples were loaded onto 8% sodium dodecyl sulfate-polyacrylamide gels. A sample was separated on a polyacrylamide gel; the procedure was abbreviated as SDS-PAGE (for sodium dodecyl sulfate polyacrylamide gel electrophoresis) according to the molecular weights. Following electrophoresis, parameters were transferred to nitrocellulose membranes (Amersham Bioscience, Piscataway, NJ, USA) using a semidry transfer apparatus (Bio-Rad, Hercules, CA, USA). The membrane was blocked with 5% (w/v) nonfat dry milk in tris-buffered saline with Tween 20 (TBST) buffer and 3% bovine serum albumin (BSA) at room temperature for 1 h to block non-specific binding sites. Subsequently, membranes were incubated with a 1:1,000 dilution of antibodies from Thermo Fisher Scientific, Inc. (Rockford, IL, USA) against rat tropomyosin receptor kinase B (TrKB; cat.# PA5-86241), sortilin (cat.# PA1-18312), phospho- phosphatidylinositol 3-kinase (pTyr458-PI3Kp85; cat.# PA5-17387), total PI3K (T-PI3K; cat.# PA5-38904), phosphor-protein kinase B (pS473-Akt; cat.# PA5-85513), T-Akt (cat.# PA5-29169), pS133-CREB (cat.# PA1-851B), and T-CREB (cat.# PA1-850), from Abcam (Cambridge, UK), phospho-glycogen synthase kinase (pS9-GSK-3β; cat.# ab131097), and T-GSK-3β (cat.#ab131356), and from Santa Cruz (CA, USA), pS33β-catenin (cat.# sc-57535) and T-β-catenin (cat.# sc-7963) for 1 h at room temperature with constant shaking. Next, membranes were probed with horseradish peroxidase-conjugated goat anti-mouse immunoglobulins (1:2000; Fluka, St. Louis, MO, USA). Eventually, the band intensity was read and analyzed using a ChemiDoc™ imaging system with Image Lab™ software version 5.1 (Bio-Rad Laboratories, Inc., Hercules, CA, USA). The results are displayed as arbitrary unit (AU) after normalization to levels of the β-actin protein.

#### 2.4.4 Histopathological Examination

Brains were carefully removed from three representative animals in different groups, rinsed with ice-cold saline, and immediately fixed with 10% formalin for 24 h. Samples were dehydrated by incubations in serial dilutions of alcohol, cleared with xylene, and embedded in paraffin at 56°C in a hot air oven for 24 h. Coronal brain sections of 4-μm thickness were processed using sledge microtome. Sections were then stained with hematoxylin and eosin (H&E) and examined under a light electric microscope (Olympus CX21, Tokyo, Japan).

### 2.5 Statistical Analysis

Data are expressed as means ± SD. Statistical analysis and graphical presentations were performed using GraphPad prism software, version 6 (GraphPad Software, Inc., San Diego, CA, USA). One-way ANOVA followed by the Tukey’s multiple comparison test was used for statistical evaluation of the difference among means, and the level of significance was set at *p* < 0.05. Data are considered outliers if the data points failed the Dixon test (Dixon, 1954) or if they exceeded four standard deviations of the mean. To ensure sample size sufficiency to establish a statistically significant difference, Mead’s “Resource Equation” was used (Mead, 1988).

## 3 Results

Noteworthy, normal group treated with Lira (NC + Lira) showed no significant changes from the normal control (NC) group in all the measured parameters; hence, all comparisons were conducted relative to NC.

### 3.1 Effect of Lira on Body Weight

**FIGURE 2:**
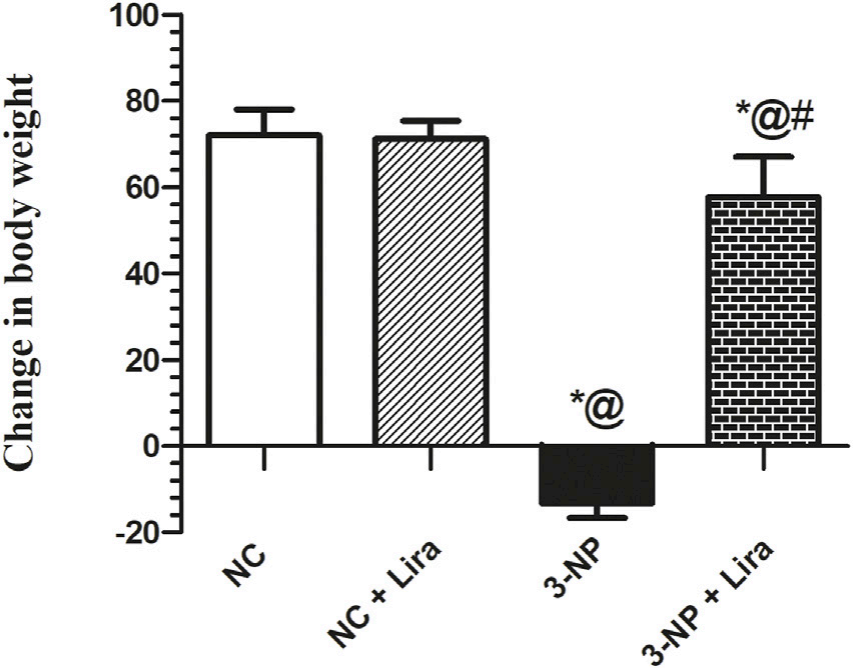
Effect of Lira treatment on body weight in the 3-NP-treated rats. Data are presented as mean ± S.D. (*n* = 15/group). Statistical analysis was performed using one-way ANOVA followed by Tukey’s multiple comparison test; compared with (*) NC, (#) 3-NP, and (@) NC + Lira groups, *p* < 0.05. ANOVA, analysis of variance; Lira, liraglutide; NC, normal control; 3-NP, 3-nitropropionic acid.

One-way ANOVA showed significant differences among groups on the percent change in body weight (Figure 2). Rats in the NC and NC + Lira showed a normal increase in body weight in the 42 days of the experiment. The 3-NP insult, however, resulted in a marked reduction in the final body weight, compared with the NC group. Treatment with Lira bolstered the body weight and obviously raised it, compared with that of 3-NP.

### 3.2 Effect of Lira on Locomotor Activity Using the Open Field Test

**FIGURE 3:**
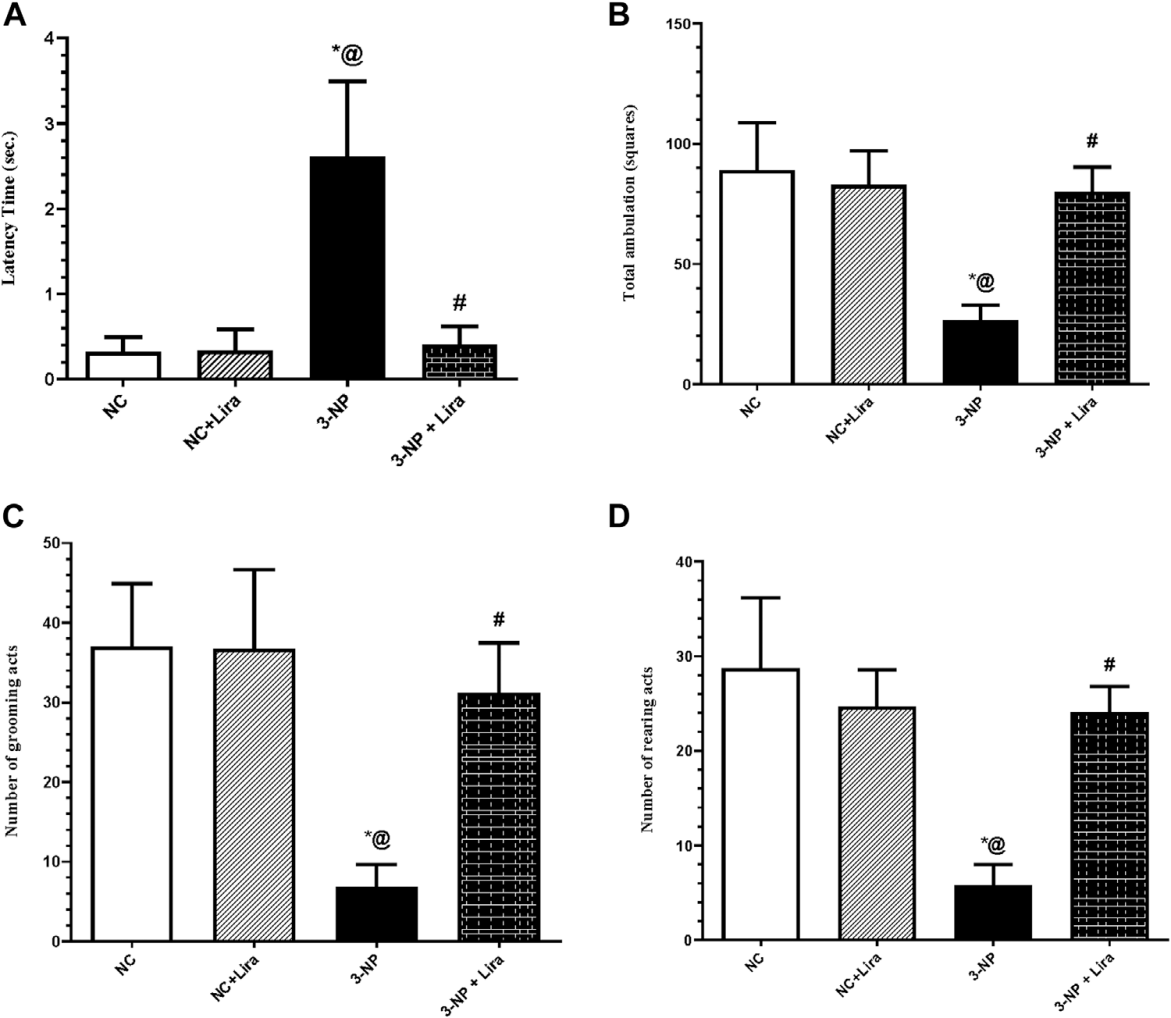
Effect of Lira treatment on the open field test (OFT) **(A)** latency, **(B)** total ambulation, **(C)** number of grooming, and **(D)** rearing acts in 3-NP treated rats. Data are presented as mean ± S.D. (*n* = 15/group). Statistical analysis was performed using one-way ANOVA followed by Tukey’s multiple comparison test; compared with (*) NC, (#) 3-NP, and (@) NC + Lira groups, *p* < 0.05. ANOVA, analysis of variance; Lira, liraglutide; NC, normal control; 3-NP, 3-nitropropionic acid; OFT. open-field test.

As depicted in Figure 3, the OFT revealed hypoactivity and gait abnormalities in 3-NP-untreated rats evidenced by a marked increase in latency time (Figure 3A) to reach approximately eightfold that of the NC group. The insult also decreased ambulation (Figure 3B), grooming (Figure 3C), and rearing (Figure 3D) frequencies by about 70%, compared with the NC group. On the other hand, treatment with Lira shortened the latency time by 92%, along with a marked increase in ambulation, grooming, and rearing frequencies to reach approximately threefold the diseased group.

### 3.3 Effect of Lira on Spatial Long-Term Memory Using the Elevated Plus Maze Test

**FIGURE 4:**
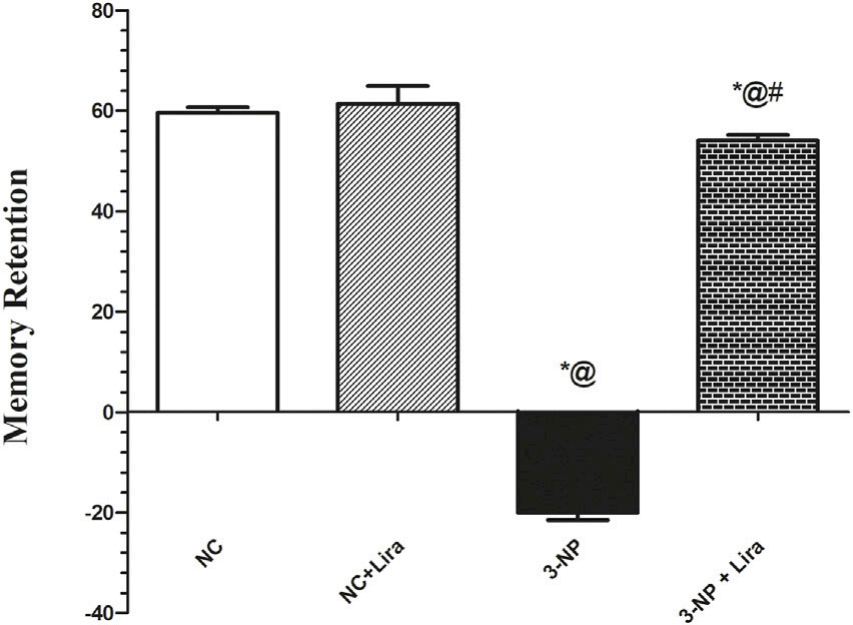
Effect of Lira treatment on the elevated plus maze (EPM). Data are presented as mean ± S.D. (*n* = 15/group). Statistical analysis was performed using one-way ANOVA followed by Tukey’s multiple comparison test; compared with (*) NC, (#) 3-NP, and (@) NC + Lira groups, *p* < 0.05. ANOVA, analysis of variance; EPM, elevated plus maze; Lira, liraglutide; NC, normal control; 3-NP, 3-nitropropionic acid.

Figure 4 shows that 3-NP-induced neurodegeneration entailed the long-term memory, where it caused a sixfold delay in the retention transfer latency compared with the vehicle treated group. In contrast, post-administration of Lira significantly abridged the retention latencies by 66% compared with the insult effect. The insult caused a marked reduction in the percentage of memory retention by 133% compared with the NC group. Contrariwise, the Lira-treated group displayed about threefold increment in this assessed parameter when compared with the 3-NP-insulted rats.

### 3.4 Lira Augments miR-130a and the Cyclic Adenosine Monophosphate/Cyclic Adenosine Monophosphate Response Element-Binding Protein/Brain-Derived Neurotrophic Factor/Tropomyosin Receptor Kinase B Trajectory

**FIGURE 5:**
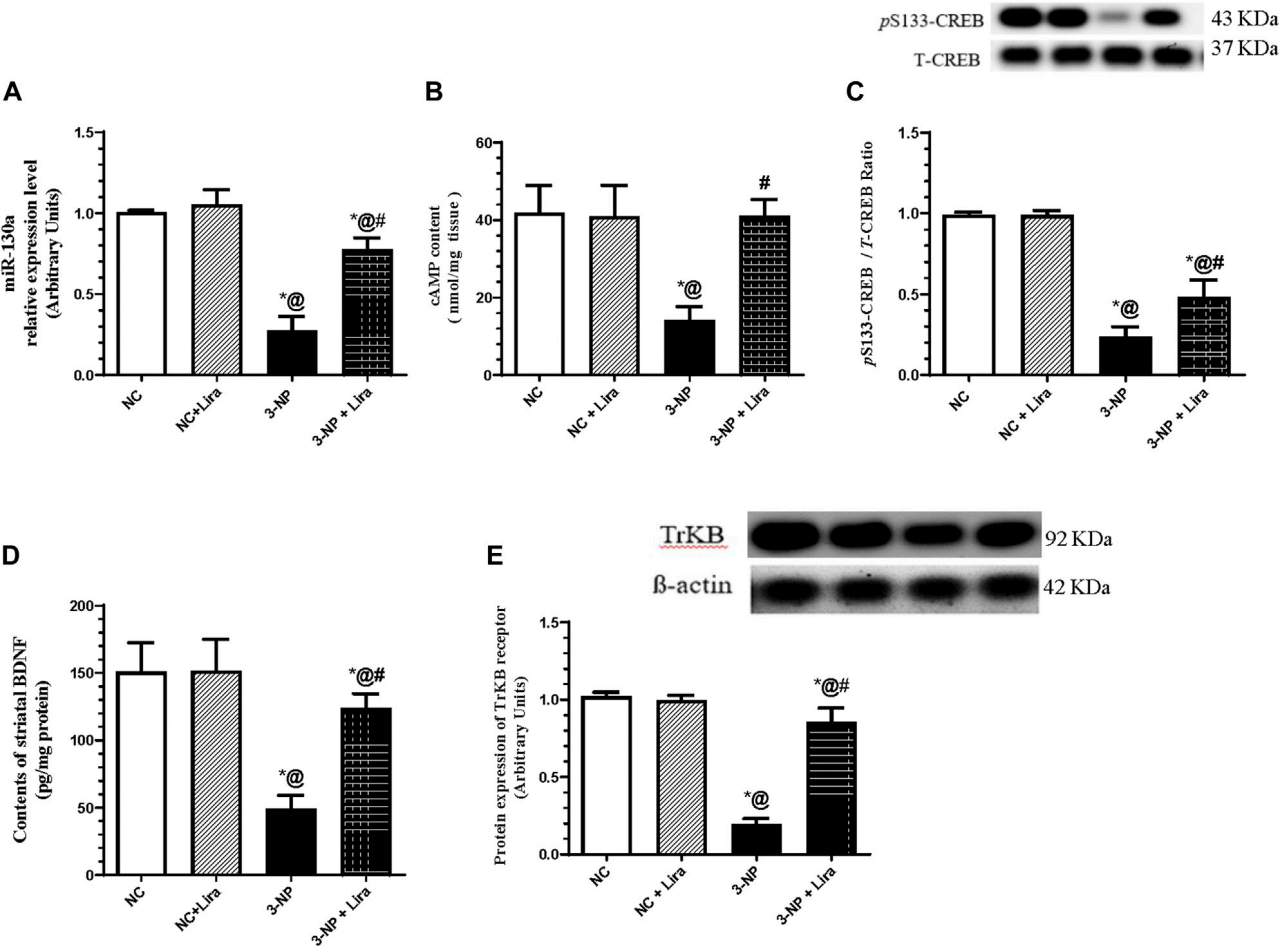
Effect of Lira on gene/protein expression and content of **(A)** miR-130a, **(B)** cAMP, **(C)** p-CREB, **(D)** BDNF, and **(E)** TrKB in 3-NP-treated rats. Data are presented as mean ± S.D. (*n* = 3–6/group). Statistical analysis was performed using one-way ANOVA followed by Tukey’s multiple comparison test; as compared with (*) NC, (#) 3-NP, and (@) NC + Lira groups, *p* < 0.05. ANOVA, analysis of variance; cAMP, cyclic adenosine monophosphate; BDNF, brain-derived neurotrophic factor; p-CREB, phosphor-cAMP response element-binding protein; Lira, liraglutide; miR, micro-RNA; NC, normal control; 3-NP, 3-nitropropionic acid; TrKB. tropomyosin-related kinase receptor.

As illustrated in Figure 5, the 3-NP administration abated the striatal gene expression of the protective gene miR-130a (Figure 5A), as well as the content of cAMP (Figure 5B), p-CREB (Figure 5C), BDNF (Figure 5D), and its receptor TrKB (Figure 5E) by about 72%, 66%, 76%, 66%, and 80%, respectively, compared with the 3-NP group. Nevertheless, rats treated with Lira augmented the neuroprotective gene and trail, where it upregulated the gene expression of miR-130a and markedly enhanced the content/protein expression of the c-AMP/CREB axis and its downstream molecules, when compared with the 3-NP group.

### 3.5 Lira Suppressed Death Receptor and Sortilin

**FIGURE 6:**
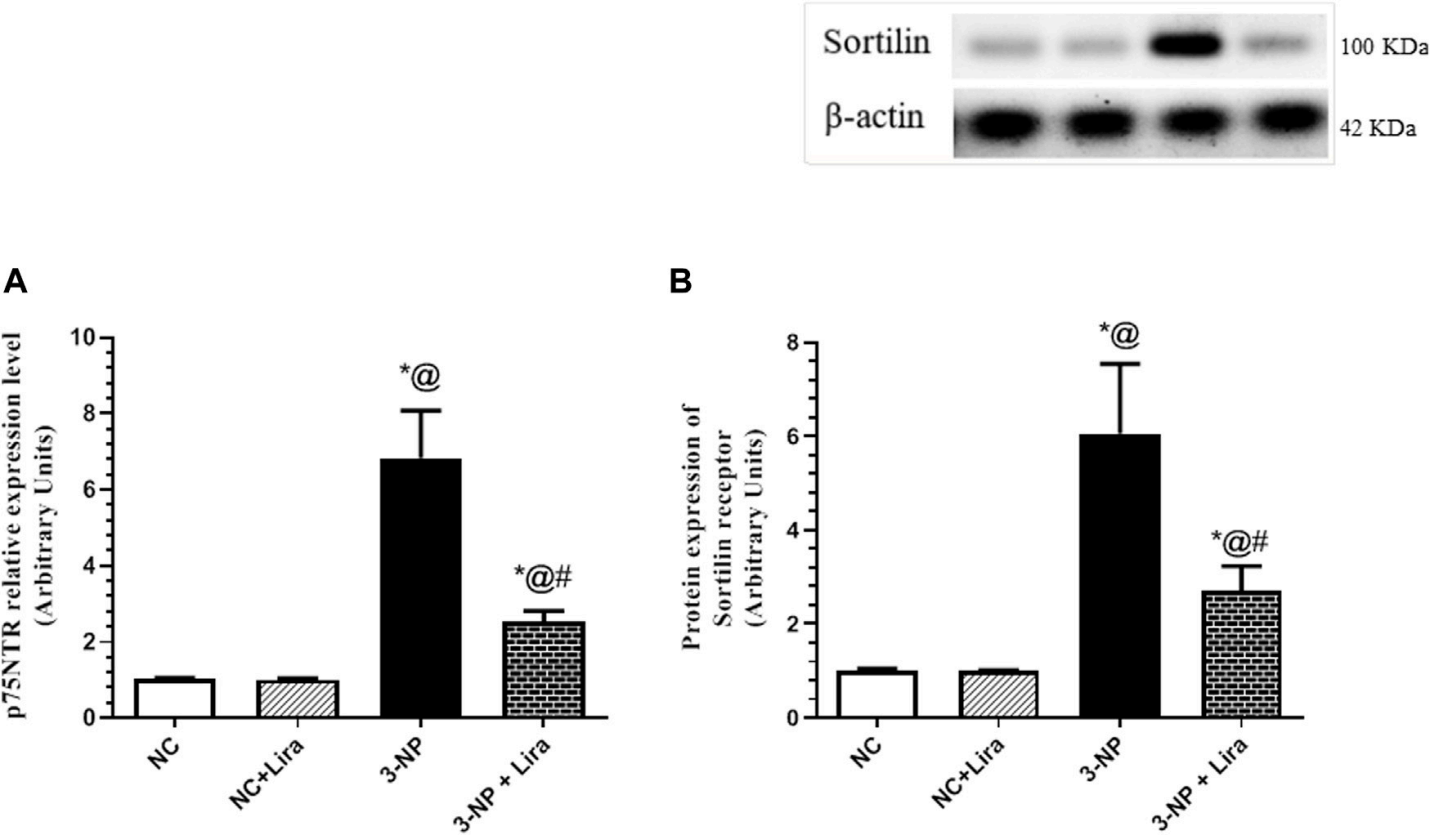
Effect of Lira on gene/protein expression of striatal **(A)** p75NTR and **(B)** sortilin in 3-NP-treated rats. Data are presented as mean ± S.D. (*n* = 3/group). Statistical analysis was performed using one-way ANOVA followed by Tukey’s multiple comparison test; compared with (*) NC, (#) 3-NP, and (@) NC + Lira groups, *p* < 0.05. ANOVA, analysis of variance; p75NTR, p75 neurotrophin receptor; Lira, liraglutide; NC, normal control; 3-NP, 3-nitropropionic acid.

As depicted in Figure 6, the 3-NP insult caused an upsurge in the mRNA expression of the death receptor, namely, p75NTR (Figure 6A), as well as the protein expression of its coreceptor sortilin (Figure 6B), mounted to about seven- and sixfold elevation, respectively, when compared with normal rats. However, Lira post-administration has softened the 3-NP effect and depressed the assessed markers to reach 37% and 45%, respectively, compared with the diseased group.

### 3.6 Lira Modulated the PI3K/Akt/GSK-3β/β-Catenin Signaling Pathway

**FIGURE 7:**
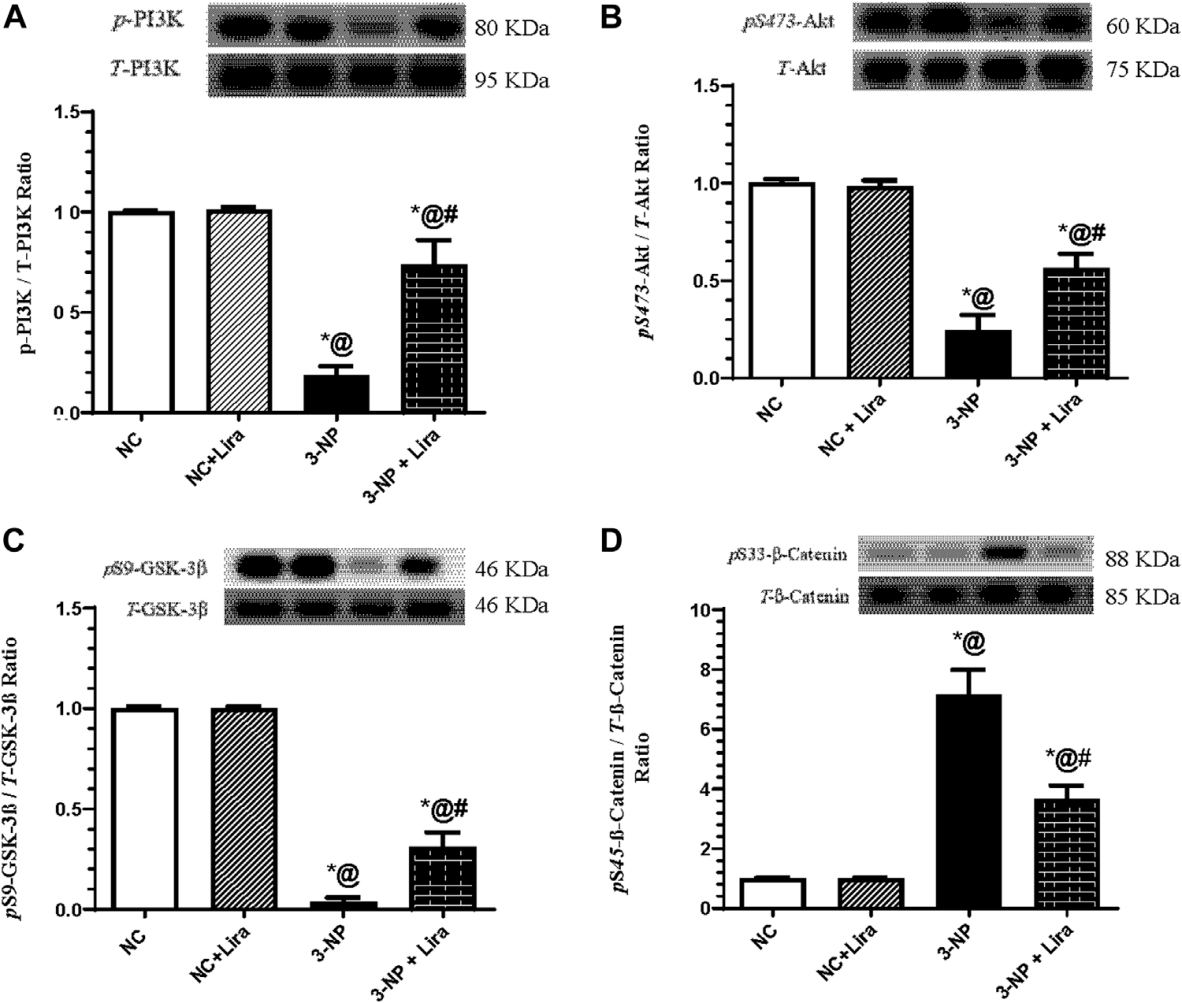
Effect of Lira on protein expression of striatal **(A)** p-PI3K, **(B)** p-Akt, **(C)** p-GSK-3*β*, and **(D)** p-*β*-catenin in 3-NP-treated rats. Data are presented as mean ± S.D. (*n* = 3/group). Statistical analysis was performed using one-way ANOVA followed by Tukey’s multiple comparison test; as compared with (*) NC, (#) 3-NP, and (@) NC + Lira groups, *p* < 0.05. ANOVA, analysis of variance; p-Akt, phosphor-protein kinase B; p-GSK-3β, phosphor=glycogen synthase kinase; Lira, liraglutide; NC, normal control; 3-NP, 3-nitropropionic acid; p-PI3K. phospho-phosphoinositide 3-kinase.

In Figure 7, the neurodegenerative effect of 3-NP was accompanied by a sharp decrease in the protein expression of p-PI3K (Figure 7A) and its downstream molecule p-Akt (Figure 7B). In turn, 3-NP activated the injurious marker GSK-3β documented by the depletion of its inactive/phosphorylated form p-GSK-3β (Figure 7C), with the subsequent phosphorylation of its downstream molecule p-β-catenin (Figure 7D) to be marked for degradation. However, administration of Lira to intoxicated rats has averted the 3-NP effects, where it turned on the protective axis and heightened the p-PI3K/p-Akt axis and inactivated p-GSK-3β by increasing its phosphorylated form, an effect that was associated by the activation/dephosphorylation of β-catenin, relative to the 3-NP insult.

### 3.7 Lira Restored Redox Balance and Decreased Neuronal Apoptosis

**FIGURE 8:**
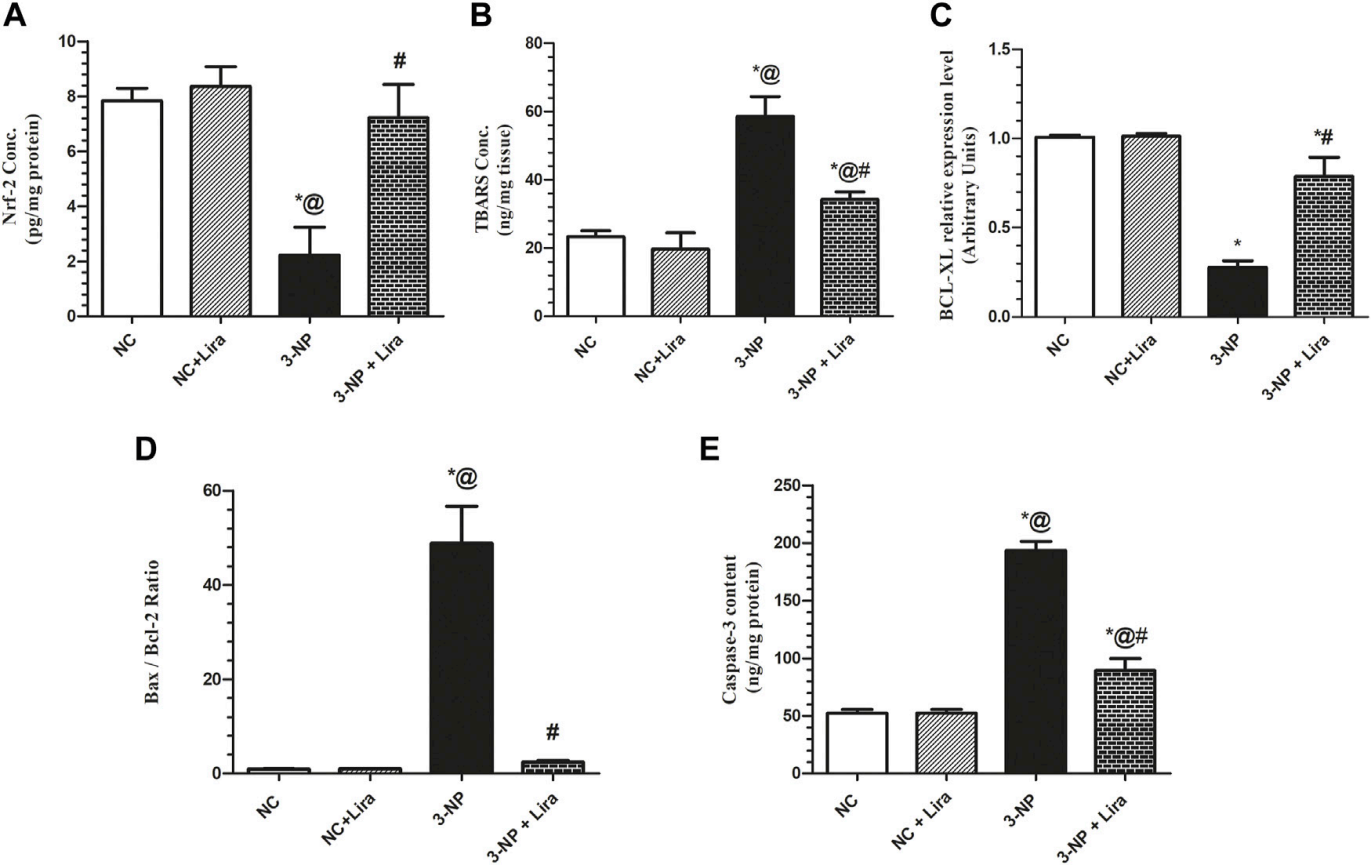
Effect of Lira on protein expression/content of striatal **(A)** Nrf-2, **(B)** TBARS, **(C)** BCL-XL, **(D)** Bax/Bcl-2 ratio, and **(E)** caspase-3 in 3-NP-treated rats. Data are presented as mean ± S.D. (*n* = 3/group). Statistical analysis was performed using one-way ANOVA followed by Tukey’s multiple comparison test; compared with (*) NC, (#) 3-NP, and (@) NC + Lira groups, *p* < 0.05. ANOVA, analysis of variance; Bax, Bcl-2-associated X protein; Bcl-2, B-cell lymphoma 2; BCL-XL, B-cell lymphoma—extra large; Lira, liraglutide; TBARS, malondialdehyde; NC, normal control; 3-NP, 3-nitropropionic acid; Nrf-2, nuclear factor E2-related factor 2.

The 3-NP-induced redox imbalance (Figure 8) was evidenced by the 72% suppression of the antioxidant transcription factor Nrf-2 (Figure 8A) effect that was accompanied by the threefold elevation of the lipid peroxidation marker TBARS (Figure 8B) compared with normal animals. Additionally, 3-NP enhanced cell apoptotic demise indicated by the 72% reduction in the antiapoptotic marker Bcl-XL (Figure 8C), with a 49-fold increase in Bax/Bcl-2 ratio (Figure 8D) and a fourfold increase in caspase-3 activity (Figure 8E), when compared with the NC group. However, Lira-treated rats displayed a remarkable antioxidant effect verified by its ability to boost striatal Nrf-2 (threefold) that was paralleled by a considerable reduction in striatal TBARS content by 41.5%, compared with the 3-NP-lesioned rats. Moreover, the antiapoptotic activity of Lira was evidenced by the reduction in Bax/Bcl-2 ratio by 95% and caspase-3 activity by 54% compared with the intoxicated group. Besides, it resulted in a threefold elevation in Bcl-XL gene expression compared with the 3-NP rats.

### 3.8 Lira obliterated Gliosis and Astrocytic Activation, but Enhanced Mature Medium Spiny Neurons

**FIGURE 9:**
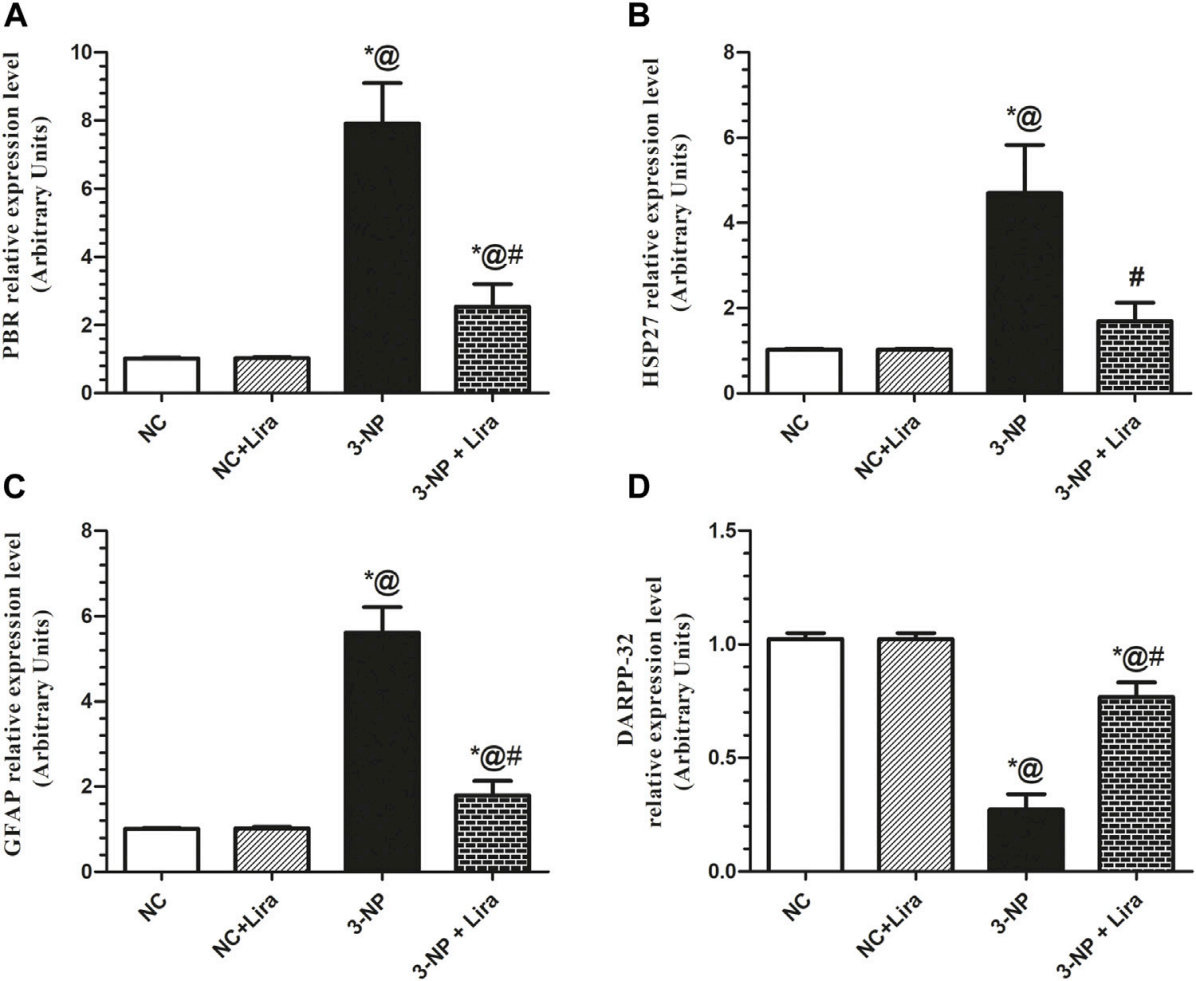
Effect of Lira on mRNA expression of striatal **(A)** PBR, **(B)** HSP27, **(C)** GFAP, and **(D)** DARPP-32 in 3-NP-treated rats. Data are presented as mean ± S.D. (*n* = 3/group). Statistical analysis was performed using one-way ANOVA followed by Tukey’s multiple comparison test; compared with (*) NC, (#) 3-NP, and (@) NC + Lira groups, *p* < 0.05. ANOVA, analysis of variance; DARPP-32, dopamine- and cAMP-regulated phosphoprotein; GFAP, glial fibrillary acidic protein; HSP27, heat shock protein 27; Lira, liraglutide; NC, normal control; 3-NP, 3-nitropropionic acid; PBR, peripheral benzodiazepine receptor.

As apparent from Figure 9, the 3-NP-intoxicated rats displayed a robust neuroinflammation, ongoing gliosis, and astrocytic activation indicated by the upregulation of the mRNA expression of PBR, HSP27, and GFAP, to about 7.8-, 4.6-, and 5.6-fold, respectively, compared with the NC group. Additionally, the toxin induced a significant neuronal damage as it caused a notable downregulation of the most widely used marker of mature MSNs, DARPP-32, by 73%, when compared with the NC group. Treatment with Lira diversely impeded the upsurge in the assessed parameters; PBR, HSP27, and GFAP reached 32%, 36%, and 32%, respectively, compared with the 3-NP group, while causing an upsurge of the striatal DARPP-32 relative expression (nearly threefold) compared with the 3-NP-lesioned rats.

### 3.9 Lira Improved Structural Alterations

**FIGURE 10:**
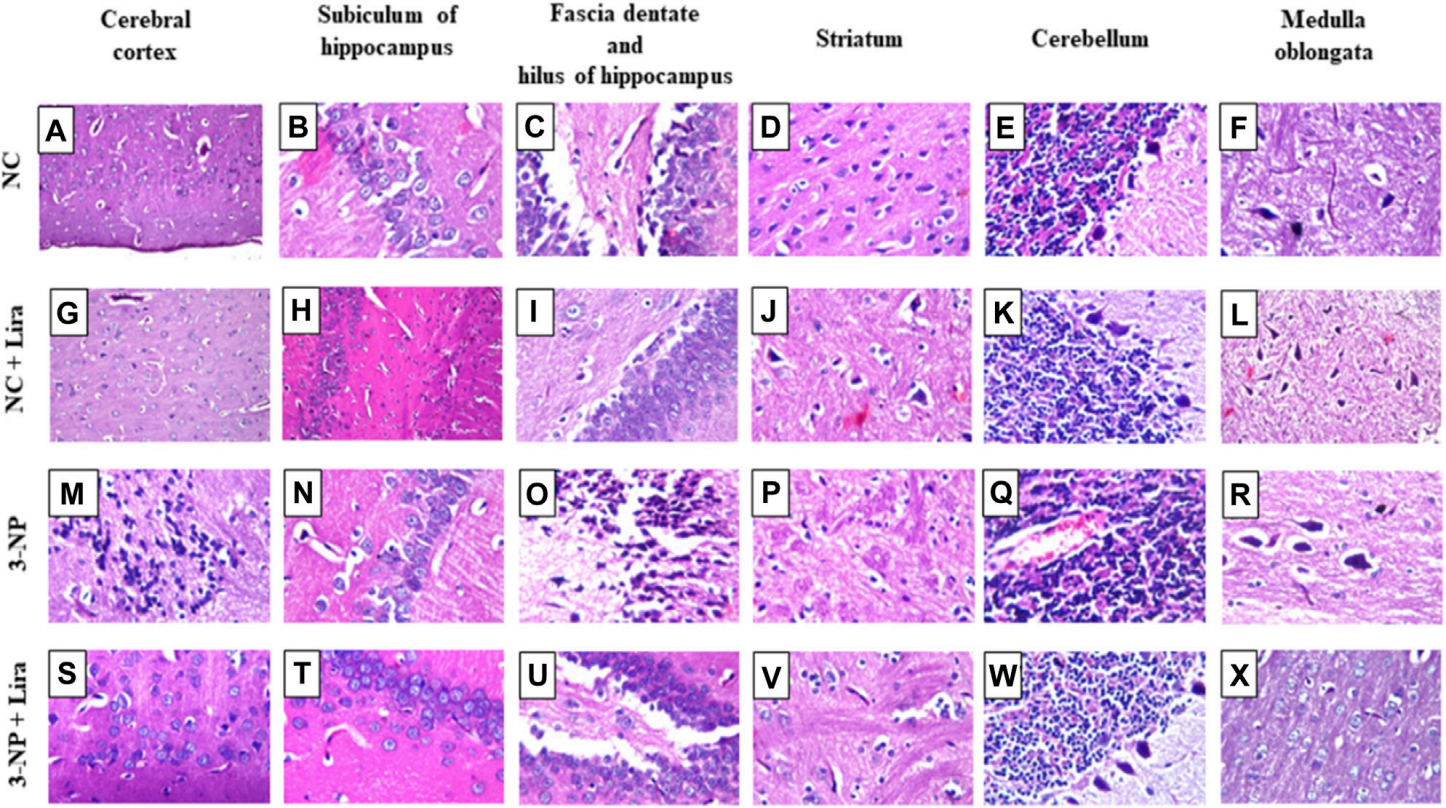
Effect of Lira on 3-NP-induced histologic alterations. Compared with the normal neuronal histological structures seen in **(A–F)** NC and **(G–L)** NC + Lira-treated group, the sections of the 3-NP-intoxicated rats show focal neuronal degeneration with gliosis in **(M)** cerebral cortex, normal morphology of **(N)** hippocampal subiculum, nuclear pyknosis and neuronal degeneration in **(O)** fascia dentate and hilus of the hippocampus, multiple focal eosinophilic plaques with focal gliosis and neuronal damage in **(P)** striatum, congestion of blood vessels in **(Q)** cerebellum, and normal morphology of **(R)** medulla oblongata. Sections of **(S–X)** 3-NP + Lira-treated group show normal histological structures of all assessed six areas (H&E × 80). Lira, liraglutide; NC, normal control; 3-NP, 3-nitropropionic acid.

As depicted in Figure 10, photomicrograph sections from NC (Figures 10A–F) and NC + Lira (Figures 10G–L) reveal normal histological structures of neurons in the cerebral cortex, subiculum of the hippocampus, fascia dentate and hilus of the hippocampus, striatum, cerebellum, and medulla oblongata. In contrast, sections of 3-NP reveal focal neuronal degeneration with gliosis in the outer area of the cerebral cortex (Figure 10M), while the inner deep area shows focal gliosis, while no histopathological alterations are seen in the subiculum of the hippocampus (Figure 10N). However, most of the neurons of the fascia dentate and hilus of the hippocampus show degeneration with nuclear pyknosis (Figure 10O). Moreover, a section of the striatum (Figure 10P) reveals multiple focal eosinophilic plaques along with diffuse gliosis in between nuclear pyknosis and neuronal degeneration. A section of the cerebellum (Figure 10Q) shows congested blood vessels; however, a section of the medulla oblongata (Figure 10R) reveals normal histological structures of neurons. On the other hand, sections of the Lira-treated group show normal histological structures of the six assessed areas (Figures 10S–X).

## 4 Discussion

The current study highlighted the potential neuro-therapeutic effect of Lira in a 3-NP-induced HD model. The findings showed that Lira prominently recovered the 3-NP-induced behavioral derangements and degeneration of striatal MSNs; the evident upturn of the neurobehavioral performance was obviously mirrored on the histopathological findings. These findings match that of a previous study, where Lira prevented hippocampal neurodegeneration and cognitive decline following intracerebroventricular injection of streptozotocin in Wistar rats (Palleria et al., 2017).

The improved functional tests have been echoed here using the OFT, which revealed shortened latency time and a noticeable increase in ambulation, grooming, and rearing frequencies in the Lira-treated rats, hindering, thus, the 3-NP effect significantly. In addition, Lira notably abridged the EPM retention latencies that were markedly delayed in the 3-NP group to display a considerable increment in the percentage of memory retention, when compared with that of the insulted rats. The ability of Lira to improve cognitive function was reported in previous models of mild traumatic brain injury (mTBI) (Li et al., 2015), Alzheimer’s disease (McClean and Hölscher, 2014), stroke (Zhu et al., 2016), and multiple sclerosis (DellaValle et al., 2016). In the current results, Lira further pinned down its capacity to improve behavioral functions by upregulating the gene expression of DARPP-32, which affects striatal function and plasticity as documented in Parkinson’s disease and schizophrenia (Meyer-Lindenberg et al., 2007), as well as models of HD. In this ailment, the protein expression of DARPP-32 was downregulated to mark the early debility of the MSNs in HD (Bibb et al., 2000; Alpaugh et al., 2017) and to concur with our results, as well.

Along with the improved behavior, the administration of Lira also ablated the 3-NP-induced histopathological alterations; the neuro-restorative impact was reflected on the improved structure that revealed normal cortical, striatal, and hippocampal morphology free of nuclear pyknosis and degeneration. Indeed, the diffuse gliosis detected in the striatum was among the alterations detected here in the present HD model to be further supported by the current augmentation of GFAP that serves as an excellent index of gliosis during neurodegeneration being the main intermediary filament of astrocytes (Brahmachari et al., 2006). However, the post-administration of Lira markedly downregulated GFAP; besides, it downregulated PBR, a hallmark of neuroinflammation that is predominantly expressed in glial cells, including astrocytes and microglia. The aptitude of Lira to abate these markers explains the inactivation of microglia that entailed rescuing striatal neurons in the current HD model. As a support to this notion, a previous study revealed that the use of a mitochondrial PBR antagonist has enhanced mitochondrial function, inactivate microglia, and reduced apoptotic neuronal death and, hence, neuroinflammation in a quinolinic acid-induced HD rat model (Ryu et al., 2005). A third marker assessed here was the HSP27, which is among the markers that increase after brain damage and is also expressed in the astroglia to indicate their activation (Renkawek et al., 1993). Akin to the other glial markers, Lira mitigated HSP27 to reach its normal level and to add to the neurotherapeutic capacity of the antidiabetic drug. These data concur with the findings of a previous study in which orphenadrine, the CNS-acting anti-muscarinic drug, has conferred *in vitro* and *in vivo* neuroprotection against 3-NP-induced neuronal damage, *via* reducing the expression of both HSP27 and PBR density in the striatum of rat brains (Pubill et al., 2001).

On the molecular level, Lira has further signified its neurotherapeutic effect by turning on the neurotrophic signaling hub cAMP/CREB/BDNF/TrKB. In the present work, Lira has enhanced the content of cAMP that was distinctly leveled off by 3-NP. This effect is a sequel of the activation of the receptor GLP-1 to concur the results in an mTBI model (Li et al., 2015). As a downstream to the latter, cAMP stimulates/activates the transcription factor CREB by phosphorylating it at Ser133 (Wang et al., 2018a) to result in a 10- to 20-fold increase in the transcriptional activity of CREB (Tanis et al., 2008), findings that consolidate the present results. In the same context, Lira was recounted to mediate its neuroprotective effect by enhancing the GLP-1 receptor-induced activation of the cAMP/CREB hub (Bao et al., 2015) using primary rat cortical astrocytes.

Upon its translocation to the nucleus, activated CREB endorses the transcription and expression of its target genes BDNF and its cognate receptor TrKB (Nair and Vaidya, 2006; Chou et al., 2020). This verity has been mirrored herein, since following the activated cAMP/p-CREB axis, Lira succeeded to oppose the effect of 3-NP and augmented the striatal contents of BDNF and the protein expression of its receptor TrKB. In turn, this points for one mechanism by which Lira has mediated its neurotrophic potential, findings that coincide with those of a previous study in a brain trauma model (DellaValle et al., 2014).

Mature BDNF (mBDNF), which is the biologically active form, binds with high affinity to the TrkB receptor and with lower affinity to the death receptor p75NTR (Kowiański et al., 2018) to mediate its beneficial effect. Notably, the mBDNF/TrKB complex is critical for structural (increases in spine density and size) and functional plasticity (LTP), and have important roles in learning and memory (De Vincenti et al., 2019). Earlier studies have highlighted the chief role of the BDNF/TrKB hub in neurogenesis, gliogenesis, neurite outgrowth, development, and maintenance of the basal ganglia in addition to promoting survival and proper function of the striatal neuronal population (Bath and Lee, 2010; Baydyuk and Xu, 2014). This pathway, hence, adds to the mechanisms by which Lira recovered cognitive and behavior functions, as well as antagonizing the 3-NP insult.

To further confine the role of BDNF in the Lira-mediated neuroprotection, the present study is the first to show that Lira has turned off the proBDNF death pathway, where it inhibited the striatal relative expression levels of the proBDNF receptors, *viz.*, p75NTR and sortilin. In agreement with the “yin–yang” hypothesis, mBDNF and its precursor, proBDNF, have opposing effects on the cellular physiology (Gibon et al., 2016). By binding to its pan-neurotrophin receptor, p75NTR, and the co-receptor, sortilin (Lee et al., 2001), proBDNF negatively regulates synaptic transmission and plasticity (Kailainathan et al., 2016). Besides, it inhibits neuronal regeneration, increases the collapse of neurite growth, and promotes apoptotic cell death, as documented here and beforehand (Liu et al., 2018). An earlier study reported that the distressing effects of proBDNF are dependent on the cellular co-expression of both p75NTR and sortilin, and suggested that neurons deficient in p75NTR are resistant to proBDNF-induced apoptosis (Teng et al., 2005). Hence, these facts affirm the present data and further explain the mechanism of Lira and nominate p75NTR to be a valuable therapeutic target (Meeker and Williams, 2014) that contributes significantly to HD progression (Plotkin and Surmeier, 2014).

The binding of mBDNF to its receptor triggers a trail of several signals that crosstalk to mediate its neurogenic effect. The first is the PI3K/Akt signaling pathway, which when turned on presents a central node for complexed functions that modulates diverse multifaceted events resulting in neuronal survival (Sánchez-Alegría et al., 2018). However, the dysregulated PI3K/Akt signaling in neurons has several harmful consequences, such as increased reactive oxygen species (ROS), membrane depolarization, mitochondrial fragmentation, as well as decreased oxidative phosphorylation and ATP production (Kim et al., 2016). In the current work, Lira has activated *p*-PI3K and *p*-Akt to concur with the findings of Yang et al. (2018) in a model of type 2 diabetes, where Lira mediated its neuroprotective effect and improved cognitive function through triggering the PI3K/Akt hub. In turn, the activated Akt plays a crucial role being a central key that manipulated several downstreams and is one of the most multifaceted kinases in the human kinome (Ahn, 2014) that ensures neuron survival (Zhang et al., 2016; Mohamed et al., 2019; Xu et al., 2020). One of the favorable effects of the activated *p*-Akt is its antioxidant (Ali et al., 2018; Wen et al., 2018) and antiapoptotic (Matsuzaki et al., 1999; Pan et al., 2019) potentials, facts that can share a part in verifying the antioxidant and antiapoptotic capacities of Lira that were documented herein.

In fact, oxidative stress (OS) and the disruption of redox homeostasis have long been held as key players in HD progression and pathogenesis (Kumar and Ratan, 2016; Li et al., 2019). The antioxidant capability of *p*-Akt can depend on the adjustment of its downstream molecules; for instance, *p*-Akt induces the expression of Nrf2, a master regulator of cellular redox homeostasis and facilitates its nuclear translocation through its tethering to the nuclear antioxidant response element (ARE). Following this union, a wide array of antioxidant enzymes is generated (Cui et al., 2016) to guard against OS (Tu et al., 2019). This has also been confirmed by previous studies that divulged that activation of the PI3K/Akt pathway induces the expression of Nrf2 to culminate in the decrease of ROS and TBARS levels, as well as neuronal apoptosis as reported here and hitherto (Ali et al., 2018; Liu et al., 2019).

Our results also highlight the role of Akt/GSK-3β/*p*-CREB and β-catenin signaling, another *p*-Akt downstream axis that reinforces its antioxidant and neurotherapeutic effects. In the Lira-treated group, the activation of Akt was tailed by the phosphorylation/inactivation of GSK-3β to concur with an earlier *in vitro* AD model (Zheng et al., 2019). The *p*-GSK-3β is a promiscuous serine-threonine kinase that regulates a wide variety of cellular functions, and its inactivation protects against neuronal toxicity (Goodenough et al., 2004). Indeed, OS brings about GSK-3β overactivation in neuronal cells (Rana and Singh, 2018), while inhibition of the latter is required to control OS in neuronal hippocampal cell lines (Jaworski et al., 2019). Moreover, inhibition of GSK-3β attenuates early stroke injury in a focal ischemic model (Wang et al., 2017). In the same milieu, Mehrafza and coworkers showed that lithium, a known GSK-3 inhibitor, has protected neurons against methamphetamine-induced neurodegeneration *via* turning on the Akt-1/GSK3 and CREB-BDNF signaling pathways, where it was recounted earlier that inactivated GSK-3β endorses the phosphorylation of CREB at Ser133 (Silva-García et al., 2018). This, in turn, allows *p*-CREB to exert its neuroprotective role as stated earlier by heightening the production of BDNF (Shimizu et al., 2019), which has an imperative role in promoting neuronal survival and differentiation by acting through its TrKB receptor. In a positive uninterrupted loop, BDNF/TrKB reactivates CREB to bestow high neuronal protection (Kowiański et al., 2018). Additionally, the crosstalk between CREB and Nrf2 via CREB-binding protein has been underlined, revealing that CREB transcription could activate the expression of Nrf2 binding partners via an indirect mechanism (Wang et al., 2016) to partake in the verification of the antioxidant capacity of Lira documented herein. These verities synchronize with the current findings, where 3-NP produced a significant reduction in *p*-Akt and, consequently, activated GSK-3β by suppressing its phosphorylation at Ser9. The inhibition of *p*-Akt entailed its downstream trajectory *p*-CREB/BDNF/TrKB to verify the role of neuronal OS in the advancement of 3-NP-induced preferential striatal degeneration, as declared herein and reported hitherto (El-Abhar et al., 2018). However, Lira post-administration resulted in offsetting the effects of 3-NP.

Aside from the GSK-3β/CREB axis, another downstream target of the GSK-3β is β-catenin, which runs inversely to its upstream GSK-3β (Huang et al., 2017). *β*-Catenin is another molecule that is known to have a dynamic role in synaptic plasticity and neurogenesis (Zhang et al., 2011; Yang et al., 2017b). Thus, one can postulate that the antioxidant effect of Lira that helps in mitigating the 3-NP neurodegenerative effect hangs on the central hub *p*-Akt/*p*-GSK-3β with its two downstream molecules *p*-CREB and β-catenin.

The ability of Lira to restore the redox balance aids in increasing cell survival, where OS is well documented to partake in apoptotic cell demise, especially the intrinsic pathway by waning mitochondrial function followed by activation of caspase-3 (Carvour et al., 2008; Méndez-Armenta et al., 2014). Besides, the ability of Lira to downregulate the expression of PBR hinders this pathway too, since as mentioned before, PBR plays a role in mitochondrial permeability transition pore formation (Jorda et al., 2005) and its decrease helps in abrogating cell death (Ryu et al., 2005).

The antiapoptotic potential of Lira also banks on the assessed pathways; indeed, earlier studies underlined the antiapoptotic role of Akt *per se* by phosphorylating the proapoptotic protein Bad, thereby, inhibiting its proapoptotic function (del Peso et al., 1997). In addition, activated Akt prohibits neuronal death effectively by inhibiting the activation of caspase-3 and Bax expression, but induces that of the antiapoptotic molecule Bcl-2, effects that were abolished upon using the specific PI3K inhibitor wortmannin (LY294002) (Matsuzaki et al., 1999). A more recent study showed that Lira protected against cerebral focal ischemia by reducing cell apoptosis *via* activating the PI3K/Akt pathway (Zhu et al., 2016). These studies harmonize with the present findings, where Lira heightened *p*-Akt and, hence, significantly reduced Bax/Bcl-2 ratio and caspase-3 activity; besides, it resulted in a substantial elevation of Bcl-XL gene expression.

Furthermore, the Akt/GSK-3β/β-catenin and CREB axes play a foremost role in cell survival *via* regulating apoptosis. The downregulation of the defense signal Akt/GSK-3β advocates the neuronal death partially *via* suppressing CREB activity, which correlates with the expression of many survival and proliferation genes (El-Abhar et al., 2018). Moreover, increased CREB activity supports cell survival by boosting the expression of cell-protective proteins, such as Bcl-2 (Cheng et al., 2016) and BDNF (Huang et al., 2015), facts that further back the present findings. Recently, a study by Zhang et al. (2019b) divulged that Lira possesses an antiapoptotic effect by enhancing *p*-CREB and upregulating the expression of the antiapoptotic Bcl-2 in an experimental diabetes model. Not only does CREB but also β-catenin represses apoptosis by inhibiting caspase-3 (Sinha et al., 2015), as well as Bax (Wang et al., 2009), while augmenting Bcl-2 (Manji et al., 2000). These data abet the present results regarding the effect of Lira on the apoptosis-related markers assessed in the present work.

Ample evidence has proclaimed that the role of miRNAs must be considered a vital aspect contributing to the pathogenesis of diverse neurodegenerative diseases as HD through targeting diverse molecules, such as BDNF (Godlewski et al., 2019). Our results showed that treatment with Lira has upregulated miR-130a noticeably, while 3-NP has markedly downregulated it, a finding that braces the neurotherapeutic effect of Lira. One clinical study has reported a marked decrease in the plasma level of miR-130a in acute ischemic stroke patients and was negatively correlated with disease risk and severity (Jin and Xing, 2018), results that may nominate it as a non-invasive promising diagnostic and prognostic biomarker (Eyileten et al., 2018). Meanwhile, the expression of miR-130a was evidently downregulated in PC12 cells after oxygen–glucose deprivation/reperfusion (OGDR) and in rats subjected to ischemic stroke and neurodegeneration. These authors proposed that not only did miR-130 offer protection against cerebral ischemic injury but also its overexpression enhanced the survival of PC12 cells, reduced apoptosis and the overproduction of ROS after OGDR, as well as the cerebral infarct volume in the stroke model. In the same setting, another study reported that miR-130a reduces the blood–brain barrier permeability induced by cerebral ischemia (Wang et al., 2018b). According to the current findings, a crosstalk between miR-130a and the assessed signals can be postulated, a notion that was partly reported in a previous study. The coresearchers revealed that the re-expression of miR-130a enhanced the activation of BDNF-mediated PI3K/Akt signaling pathway by inhibiting PTEN, a mechanism that underlies the neuroprotection against cerebral ischemia–reperfusion injury. However, the correlation between miR-130a and the BDNF/TrKB/PI3K/Akt/GSK-3β/β-catenin/CREB trajectory, as well as the p75NTR–sortilin receptor complex along with the redox imbalance and apoptosis has been reported first in the present work.

In conclusion, the current study has revealed the promising neurotherapeutic potential of Lira against 3-NP-induced HD *via* modulating the crosstalk between cAMP/CREB/BDNF/TrkB, PI3K/Akt/GSK-3β/β-catenin, and GSK-3β/CREB trajectories. In addition, Lira upregulated the newly diagnostic non-invasive promising biomarker, miR-130a, while it curbed the p75NTR–sortilin receptor complex. Meanwhile, Lira purveyed pronounced antioxidant and antiapoptotic potentials in 3-NP model of HD. These mechanisms resulted in an upturn in behavioral and morphological results.

## 5 Limitations and strengths

The strength of our work is studying the therapeutic effect of the antidiabetic drug Lira against 3-NP-induced HD-like model. The effect was proven through improving cognition and memory, and the upturn of the striatal morphological structure that was altered in the 3-NP-untreated group. On the molecular level, the drug enhanced several survival pathways and abated others that play a role in neurodegeneration.

The limitations of the possible repurposing of Lira is using only one HD model; however, this effect should be attested upon using other HD models. Also, the study use male rats only, and the effect of Lira should also be carried out on different species and genders. Moreover, other signaling pathways have to be evaluated to unveil more therapeutic mechanisms before proceeding to the clinical trials.

## Data Availability

The raw data supporting the conclusion of this article will be made available by the authors, without undue reservation.
