# Supplementary figures and images for: Liraglutide Improves Cognitive and Neuronal Function in 3-NP Rat Model of Huntington’s Disease
Source: Front Pharmacol. 2021 Dec 22;12:731483. doi: 10.3389/fphar.2021.731483 (PMC8727874; doi:10.3389/fphar.2021.731483)

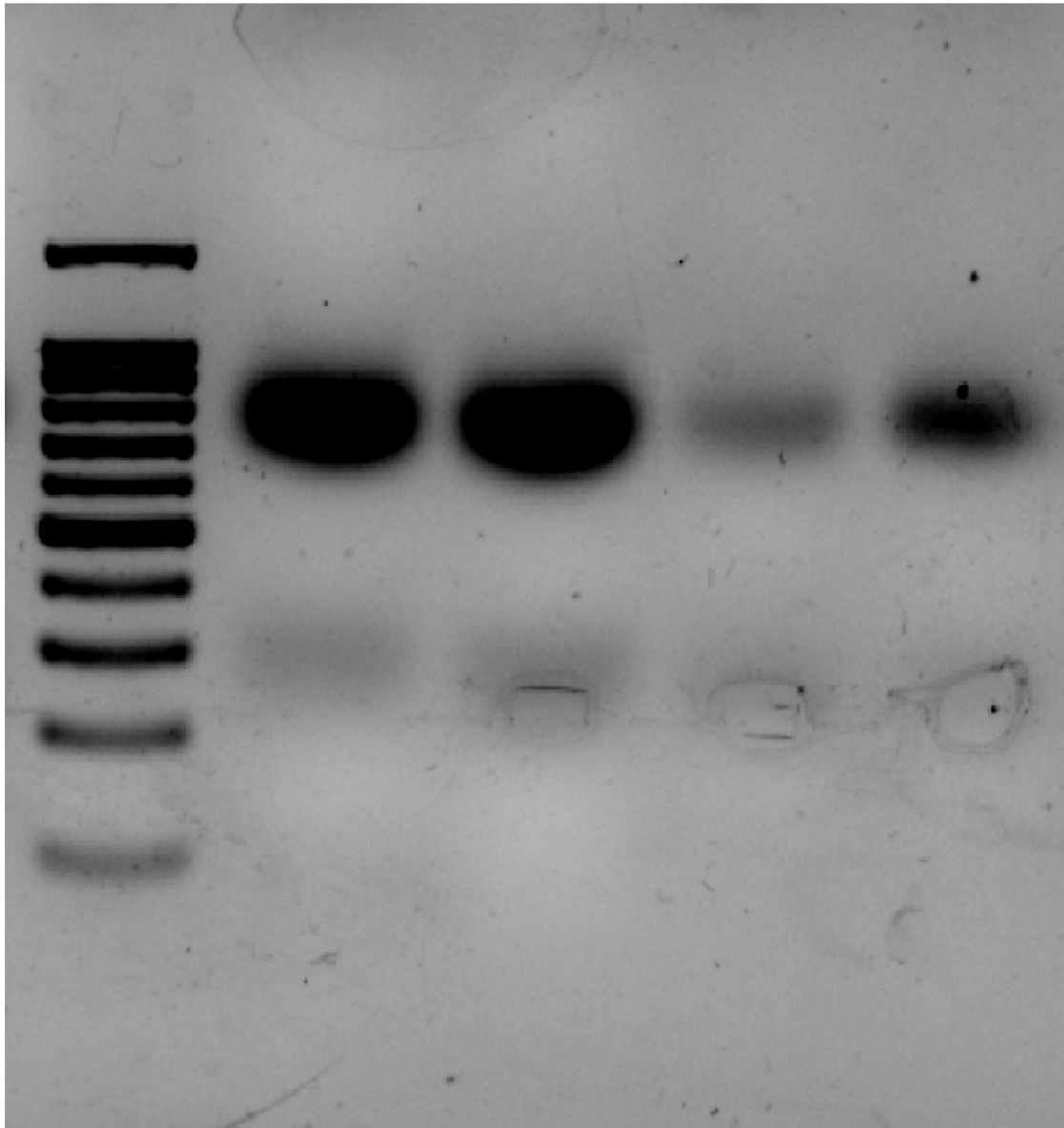

Beta Catenin

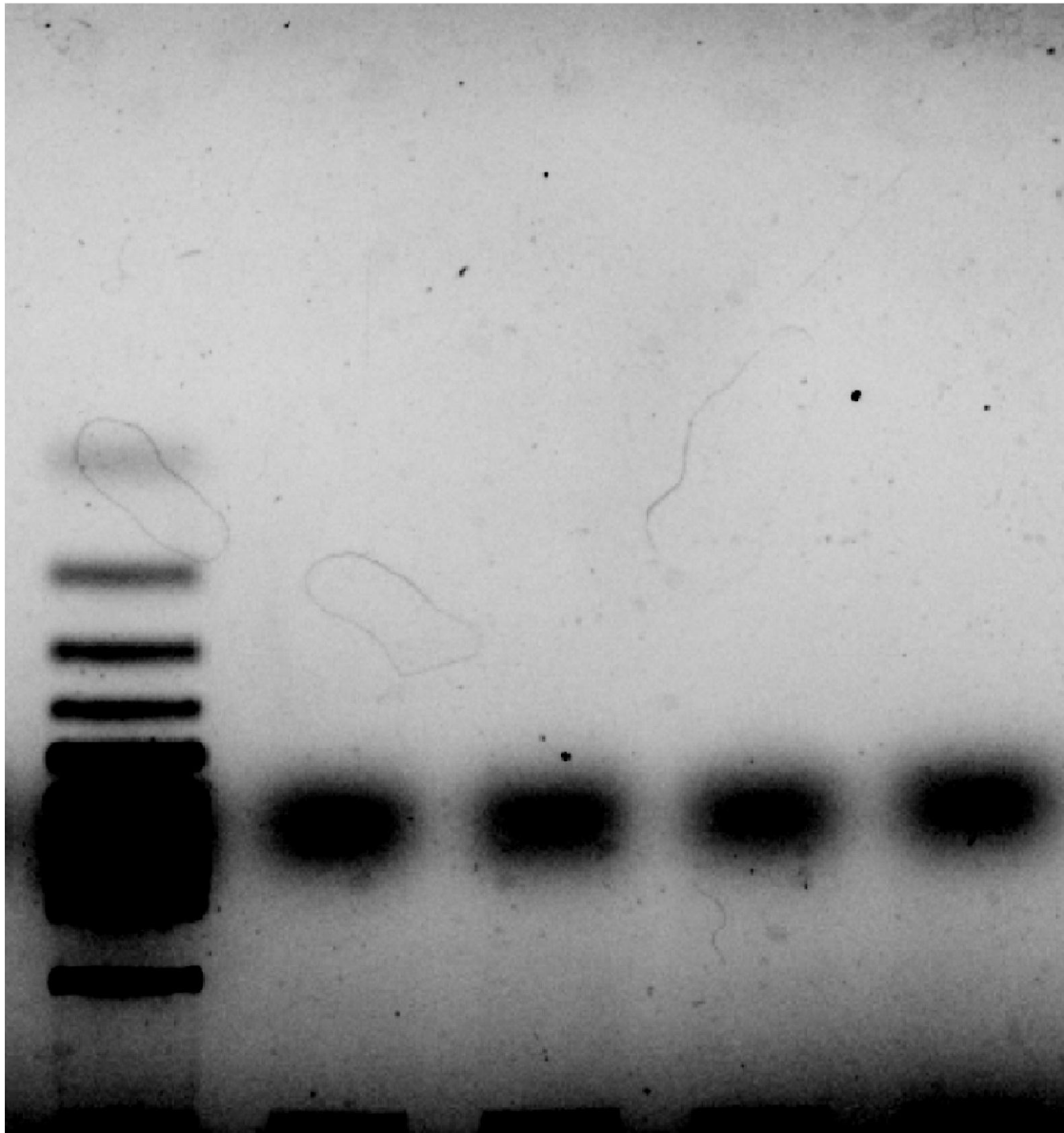

Beta actin

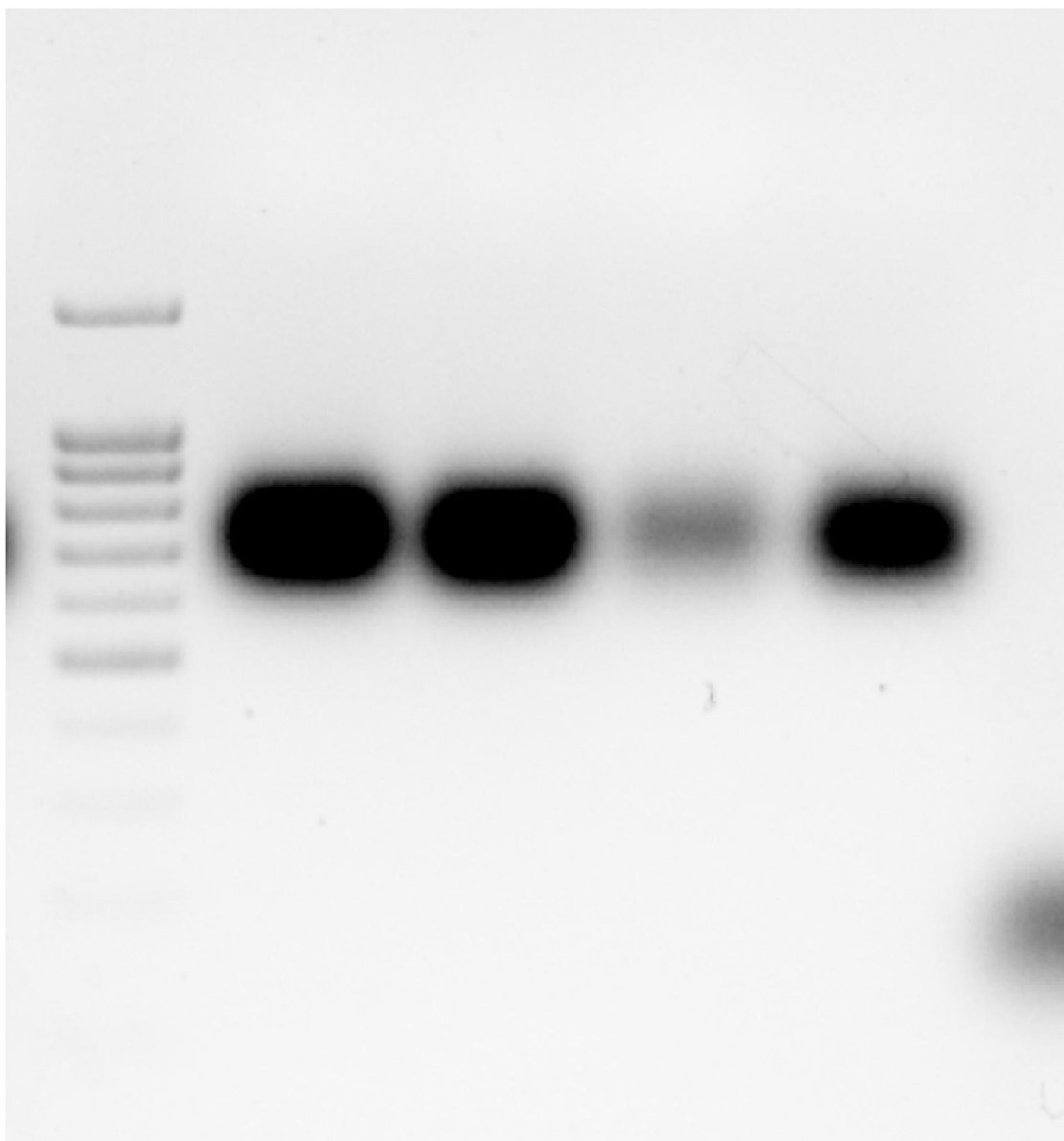

p CREB

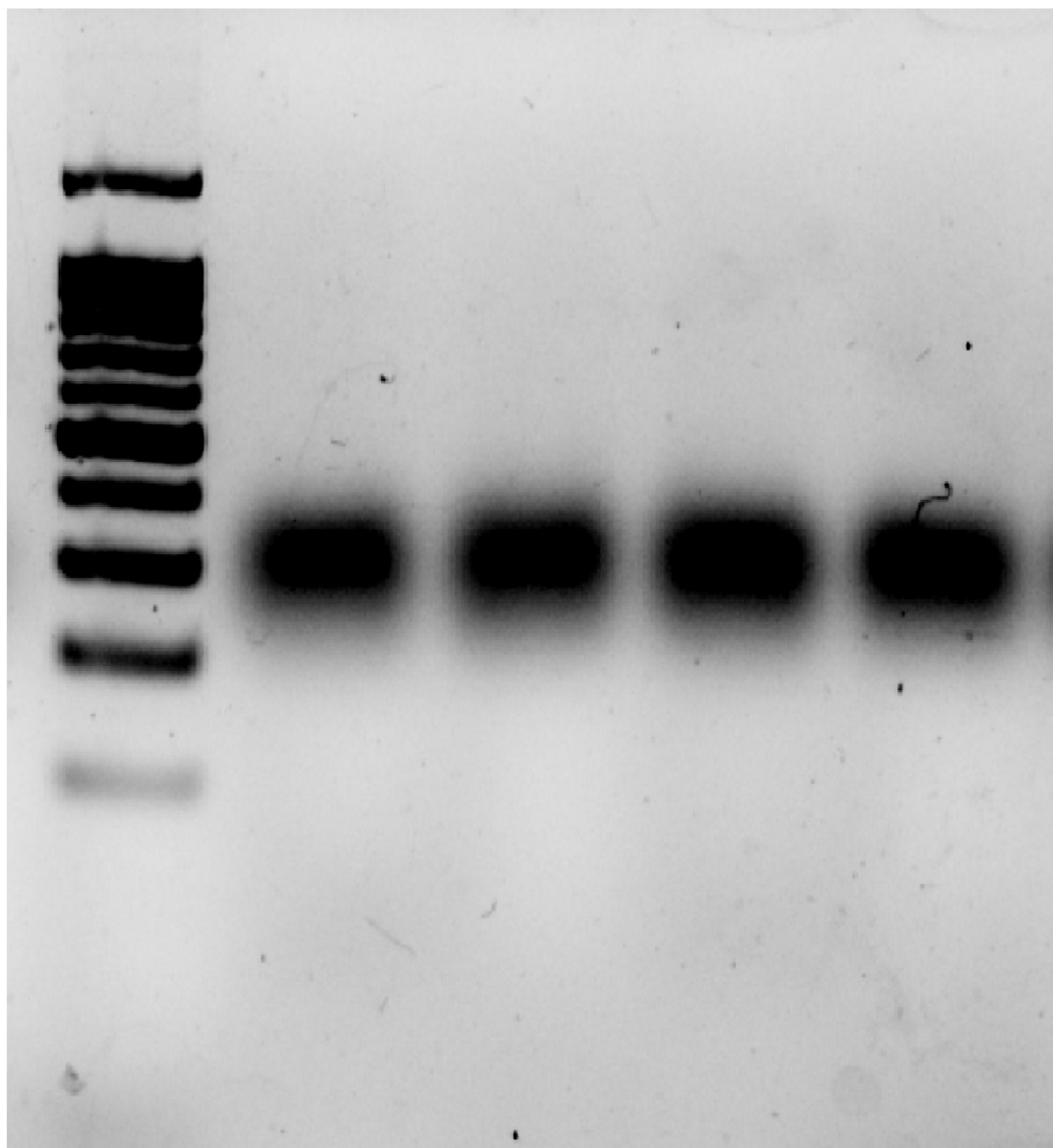

T CREB

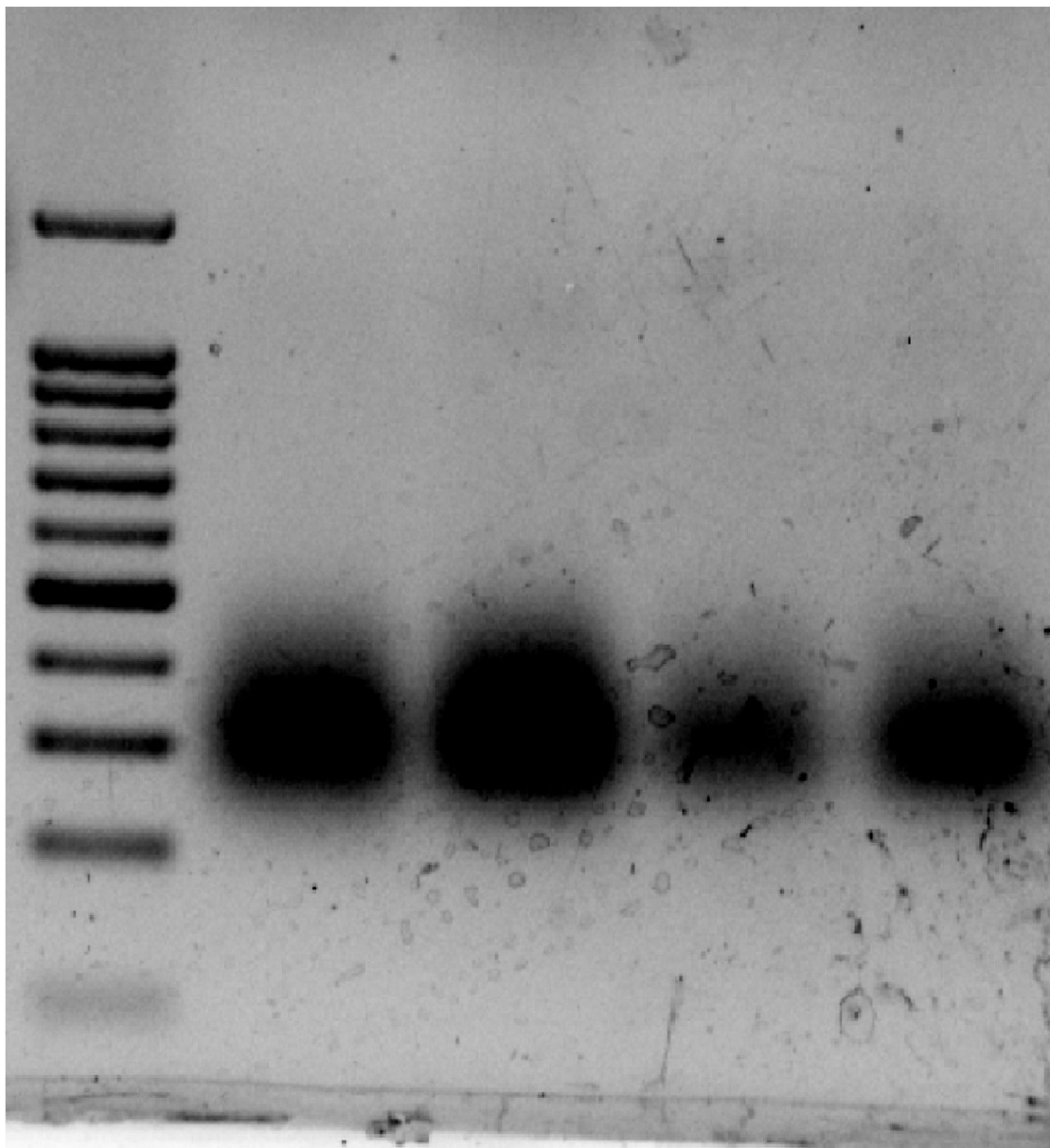

p AKt

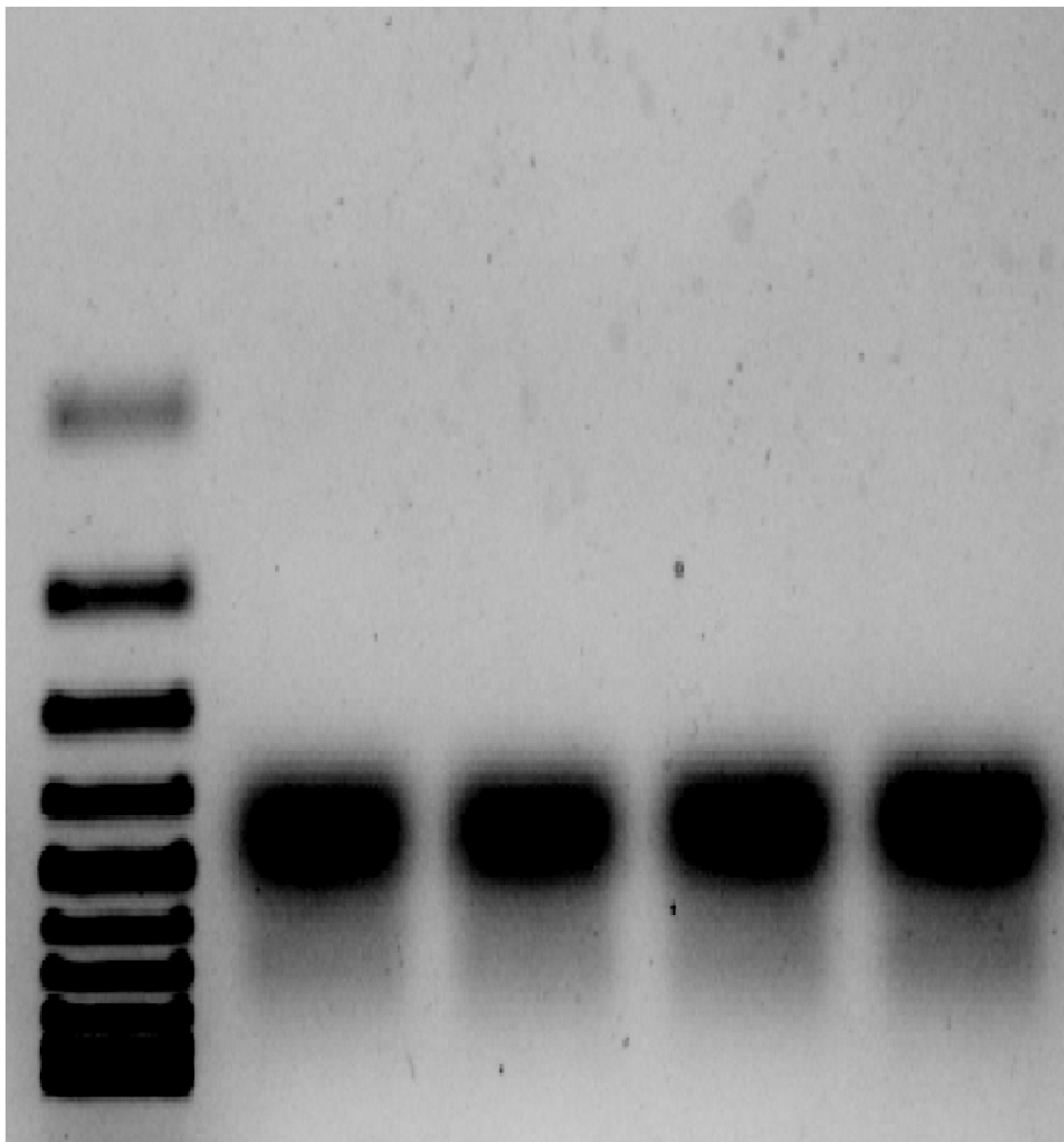

T Akt

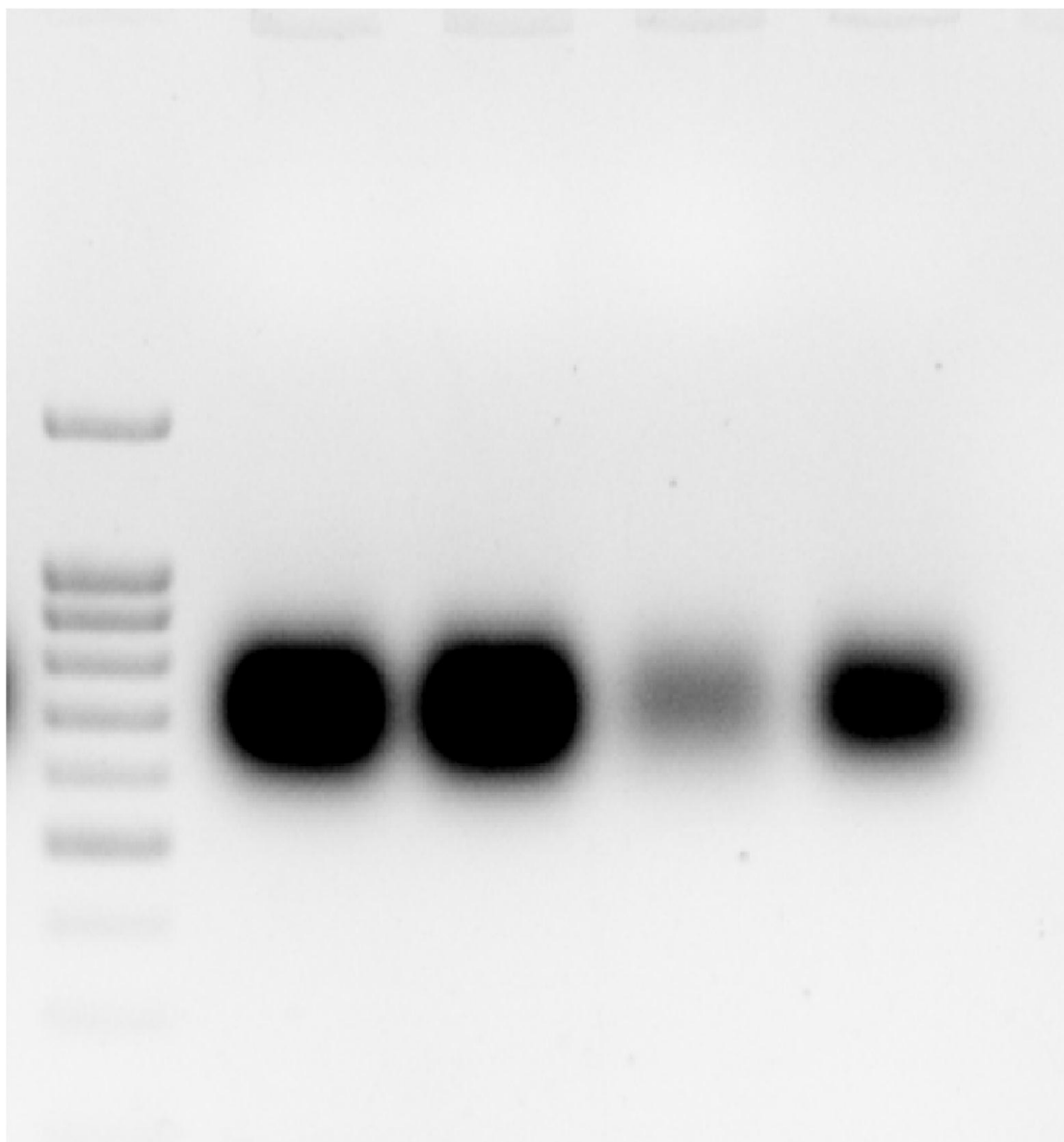

pGSK

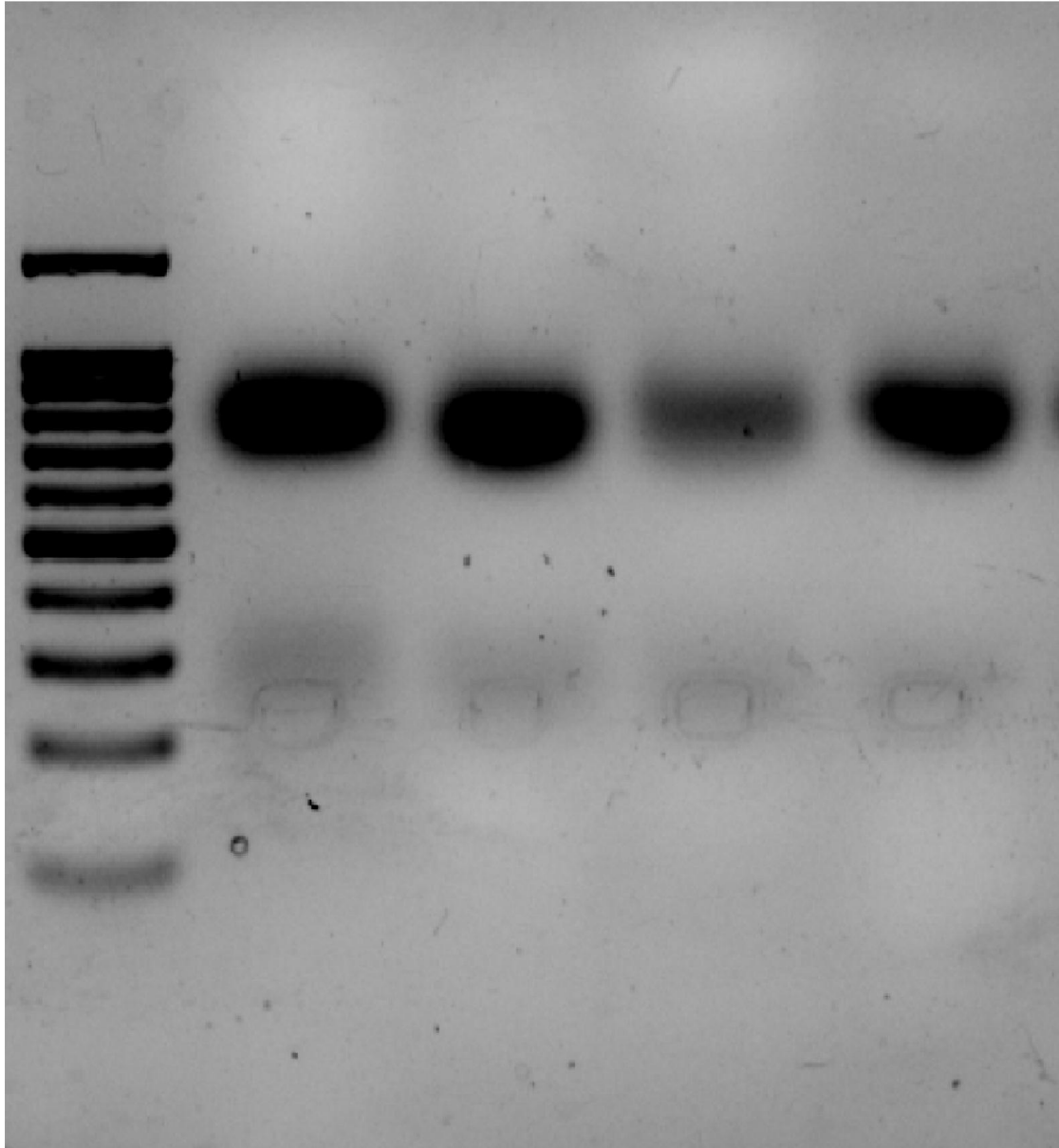

p PI3K

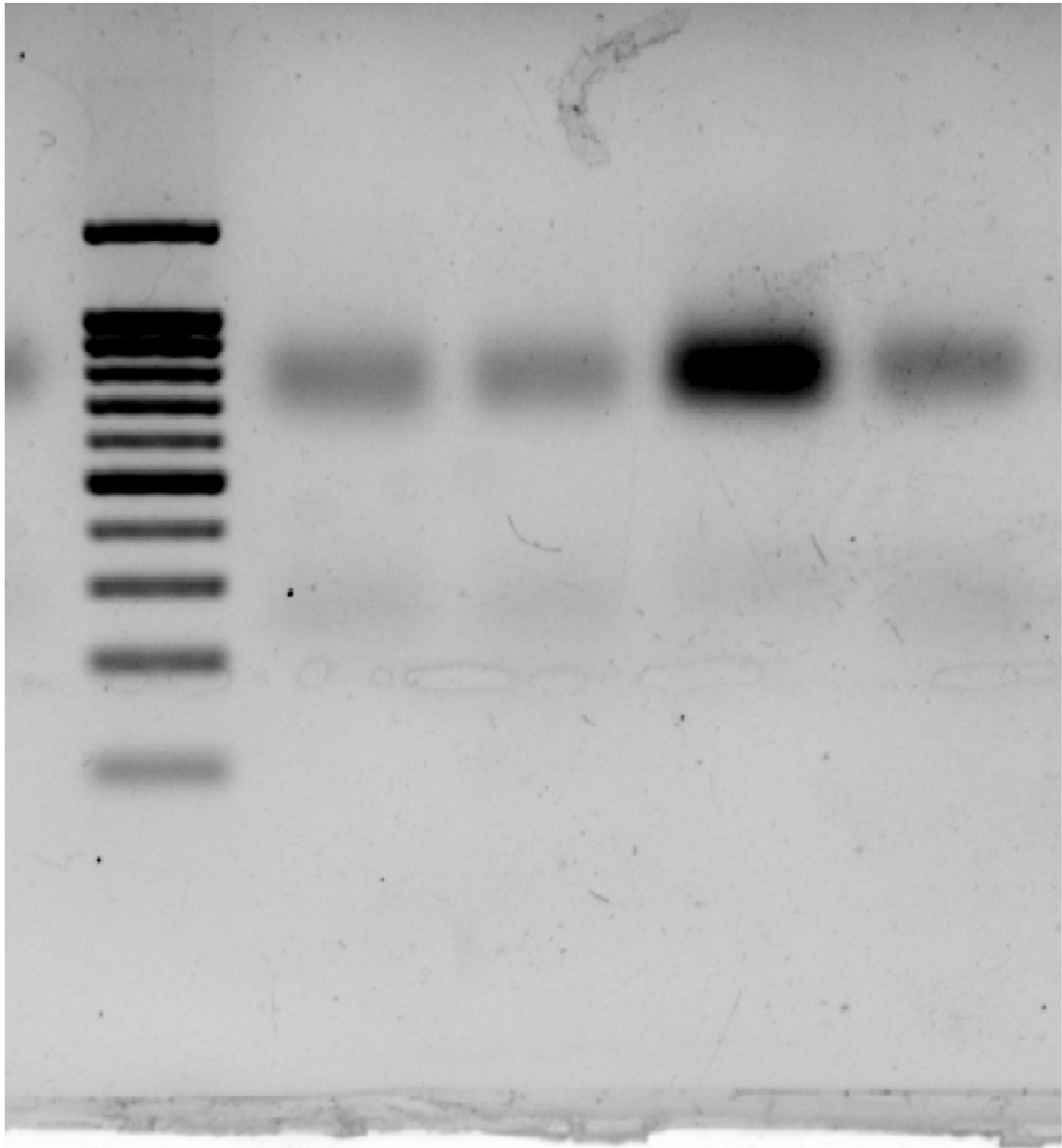

Sortilin

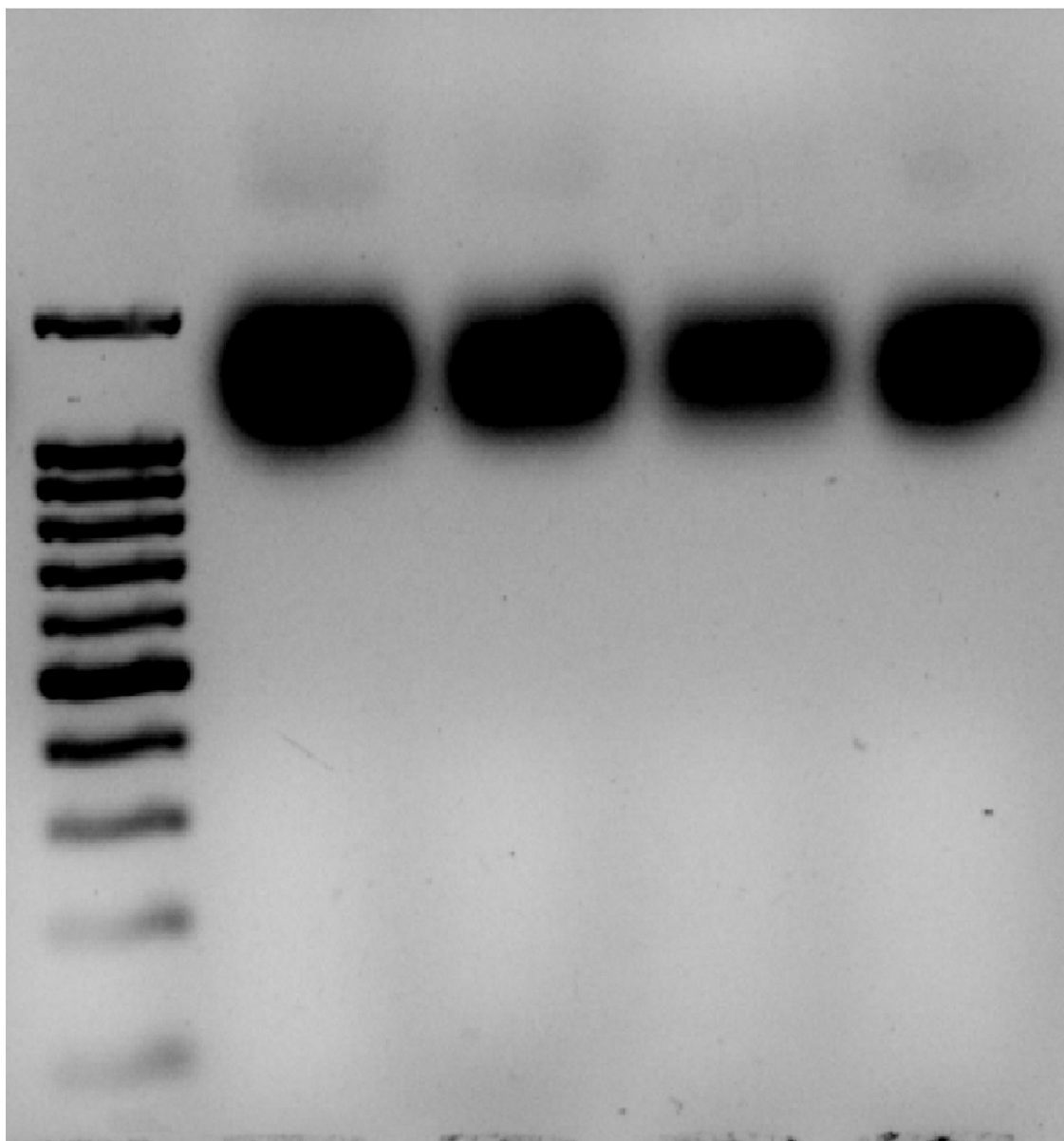

TrKB

Supplement: Supplementary file 1 [file DataSheet2.PDF]

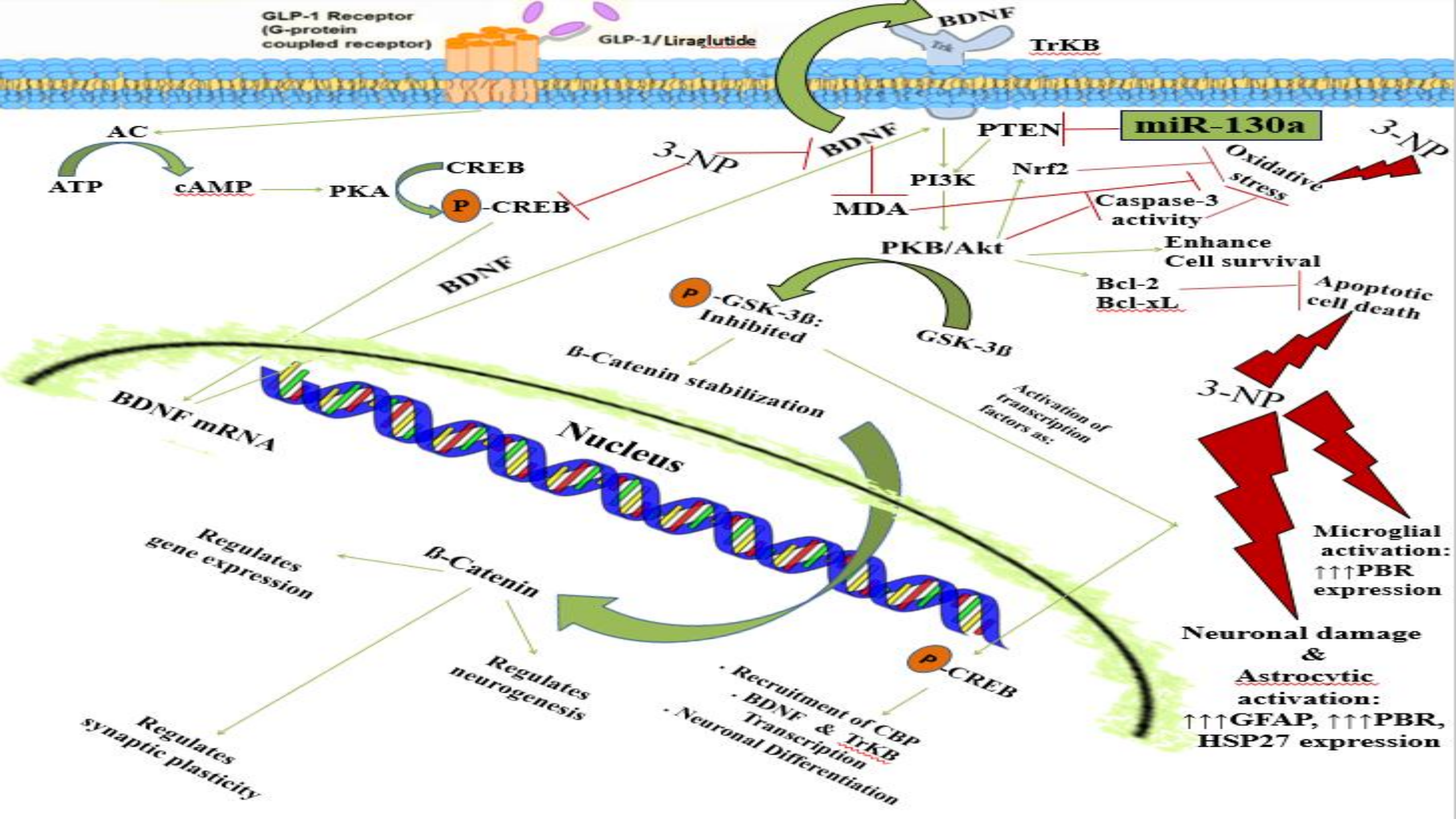

Supplement: Supplementary file 3 [file DataSheet1.PDF]
