# Supplementary material for: Liraglutide Improves Cognitive and Neuronal Function in 3-NP Rat Model of Huntington’s Disease
Source: Front Pharmacol. 2021 Dec 22;12:731483. doi: 10.3389/fphar.2021.731483 (PMC8727874; doi:10.3389/fphar.2021.731483)
Supplement: Supplementary file 2 [file DataSheet3.PDF]

Parameters / Groups	**Change in body weight**	**Memory Retention**	**cAMP**	***p*S133-CREB**	**BDNF**	**TrKB**
NC	100 ± 8.37	100 ± 1.73	100 ± 16.88	100 ± 1.5	100 ± 12.43	100 ± 2.65
NC + Lira	98.83 ± 5.87	102.95 ± 5.92	98.14 ± 18.92	99.63 ± 2.97	98.24 ± 10.98	97.39 ± 3.18
3-NP	-18.36 ± 4.76$^{*@}$	-33.57 ± 2.47$^{*@}$	34.07 ± 8.18$^{*@}$	23.93 ± 6.16$^{*@}$	33.54 ± 2.65$^{*@}$	19.43 ± 3.15$^{*@}$
3-NP + Lira	80.06 ± 12.99$^{*@\#}$	90.66 ± 1.84$^{*@\#}$	98.4 ± 9.78$^{\#}$	48.61 ± 10.63$^{*@\#}$	101.44 ± 3.74$^{*@\#}$	83.82 ± 8.58$^{*@\#}$

Parameters / Groups	**p75NTR**	**Sortilin**	***p*-PI3K**	***p*-Akt**	***p*-GSK-3β**	***p*-β-catenin**
NC	100 ± 2.6	100 ± 2.48	100 ± 0.7	100 ± 1.76	100 ± 0.74	100 ± 1.47
NC + Lira	99.12 ± 2.15	98.73 ± 0.87	101 ± 1.38	98.01 ± 2.96	99.9 ± 0.88	100 ± 1.56
3-NP	667.94 ± 121.11$^{*@}$	593.14 ± 145.25$^{*@}$	18.69 ± 4.45$^{*@}$	24.38 ± 7.8$^{*@}$	3.86 ± 1.95$^{*@}$	707.31 ± 81.51$^{*@}$
3-NP + Lira	248.09 ± 27.12$^{*@\#}$	264.05 ± 52.58$^{*@\#}$	73.78± 12.18$^{*@\#}$	55.97 ± 7.46$^{*@\#}$	31.12 ± 7.14$^{*@\#}$	361.6 ± 45.37$^{*@\#}$

Parameters / Groups	**Nrf-2**	**TBARS**	**Bax/Bcl-2 ratio**	**BCL-XL**	**Caspase-3**	**Bax/Bcl-2 ratio**
NC	100 ± 5.82	100 ± 0.08	100 ± 3.33	100 ± 1.16	100 ± 5.50	100 ± 3.33
NC + Lira	106.7 ± 9.15	84.39 ± 20.54	101.62 ± 2.59	100.50 ± 1.57	110.28 ± 5.93	101.62 ± 2.59

3-NP	28.39 ± 12.94$^{*@}$	250.99 ± 25.08$^{*@}$	4931.31 ± 795.56$^{*@}$	27.53 ± 3.66*	371.61 ± 16.35$^{*@}$	4931.31 ± 795.56$^{*@}$
3-NP + Lira	92.18 ± 15.46$^{\#}$	146.78 ± 9.18$^{*@\#}$	243.33 ± 41.00$^{\#}$	78.12 ± 10.69$^{*\#}$	171.63 ± 21.09$^{*@\#}$	243.33 ± 41.00$^{\#}$

**Parameters / Groups**	**miR-130a**	**PBR**	**HSP27**	**GFAP**	**DARPP-32**
NC	100 ± 1.03	100 ± 2.08	100 ± 2.57	100 ± 1.30	100 ± 2.45
NC + Lira	104.66 ± 8.92	100.98 ± 2.50	100 ± 2.77	100.89 ± 3.53	99.9 ± 2.60
3-NP	27.28 ± 8.70$^{*@}$	777.31 ± 115.72$^{*@}$	460.78 ± 110.78$^{*@}$	555.74 ± 59.28$^{*@}$	26.61 ± 6.60$^{*@}$
3-NP + Lira	77.01 ± 7.15$^{*@\#}$	249.31 ± 64.67$^{*@\#}$	165.59 ± 42.81$^{\#}$	177.52 ± 34.04$^{*@\#}$	75.08 ± 6.19$^{*@\#}$
